# Excess hospitalizations and in-hospital mortality associated with seasonal influenza in Italy: a 11-year retrospective study

**DOI:** 10.1186/s12879-024-09071-z

**Published:** 2024-02-20

**Authors:** Giovanni Fattore, Benedetta Pongiglione, Luigi Vezzosi

**Affiliations:** 1 Department of Social and Political Sciences, Bocconi University, Milan, Italy; 2 SDA Bocconi School of Management, Centre for Research on Health and Social Care Management, Milan, Italy; 3 Department of Hygiene and Health Prevention, Prevention of Infectious Diseases Unit, Health Protection Agency Val Padana, Mantua, Italy

**Keywords:** Influenza, Burden, Excess, Hospitalization, Cardiovascular, Respiratory, Italy

## Abstract

**Background:**

Influenza and flu-like syndromes are difficult to monitor because the symptoms are not specific, laboratory tests are not routinely performed, and diagnosis codes are often lacking or incompletely registered in medical records. This may result in an underestimation of hospital admissions, associated costs, and in-hospital mortality. Therefore, this study aimed to estimate the public health and economic burden of hospitalisations associated with influenza in Italy, at the national and regional levels.

**Methods:**

This 11-year retrospective study included patients admitted to hospitals for influenza or diagnoses associated with influenza (including respiratory and cardiocirculatory conditions) from 2008/09 to 2018/19. Data on hospitalisations were extracted from the Italian Hospital Discharge Records. Information on weekly influenza-like syndrome incidence and weekly average temperature were used to estimate the burden of influenza in terms of hospital admissions in every Italian region and for different age groups by applying a negative binomial model. The model was also applied to estimate in-hospital mortality and the total costs of influenza and influenza-like hospital admissions.

**Results:**

Over the study period, in addition to 3,970 average seasonal admissions coded as influenza, we estimated an average of 21,500 excess hospitalization associated with influenza per season, which corresponds to 36.4 cases per 100,000. Most of the excess hospitalisations concerned older individuals (> 65 years) and children (0–4 years) with 86 and 125 cases per 100,000, respectively. Large variations were observed across regions. Overall, the total estimated hospital burden associated with influenza (including respiratory and cardiocirculatory conditions) was approximately €123 m per year. While the in-hospital mortality for admissions with a primary diagnosis of influenza was very low (~ 150 cases per season), cases increased dramatically for primary diagnoses of influenza and pneumonia (about 9,500 cases per season). The average seasonal in-hospital deaths attributable to influenza were equal to 2,775 cases.

**Conclusions:**

Our findings suggest a remarkable underestimation of the burden of influenza, mostly in the older population but not neglectable in younger individuals. Our results may aid the management of current and future flu seasons and should be used for policy making (e.g., vaccine strategies) and operation management choices (e.g., planning and staffing beds during influenza peaks). Overall, the present study supports the need for increased testing for influenza in Italy to tackle the current underestimation of influenza burden.

**Supplementary Information:**

The online version contains supplementary material available at 10.1186/s12879-024-09071-z.

## Background

Influenza is an acute viral respiratory infectious disease, the main symptoms of which are inflammation of the nasal mucosa, pharynx and conjunctiva, headache, and severe and often generalized myalgia. In the Northern hemisphere, the peak of the flu season is usually observed in the winter months with outbreaks occurring annually, causing significant morbidity and even mortality in cases of severe clinical complications [1]. The World Health Organization (WHO) has estimated that, worldwide, the flu virus causes approximately 3–5 million serious cases annually and 290,000–650,000 deaths [2]. A recent Italian study estimated the excess deaths attributable to influenza epidemics to be approximately 7,000, 20,000, 16,000, and 25,000 in the 2013/14, 2014/15, 2015/16, and 2016/17 seasons, respectively, with most of the influenza-associated deaths per year registered among the older population [3]. With the arrival of COVID-19, in 2020, respiratory infections ranked among the top 10 causes of death in Italy, with a 211% increase in deaths in March–April 2020 compared to the average of the preceding five years [4]. From an economic viewpoint, influenza causes high consumption of healthcare resources due to hospitalisations, medical visits, and the use of drugs [5–7], sometimes in inappropriate ways [8]. In addition, influenza has a crucial impact on productivity due to absenteeism and presenteeism [9].

The most common complications associated with the flu are generally related to the respiratory system; however, other body systems can also be affected by the virus. For example, some cardiovascular complications, such as myocardial infarction, have been associated with previous respiratory infections, including influenza [10, 11]. It has also been shown that vaccination against the influenza virus in older individuals leads to a reduction in the risk of hospitalisation (by approximately 20%) for heart and cardiovascular diseases [12]. A recent study in Alberta (Canada) found that recent influenza vaccination significantly reduced the hazard of stroke [13]. In addition, complications may arise from the exacerbation of pre-existing chronic diseases, such as asthma, chronic obstructive pulmonary disease [14, 15], diabetes [16, 17], and cardiovascular diseases [18].

In Italy, the Ministry of Health annually issues recommendations for the prevention and control of seasonal influenza. In the latest document, season 2022/23, subjects at high risk of influenza-related complications or hospitalisations are advised to be vaccinated. They included individuals of any age suffering from chronic conditions, such as diabetes and cardiovascular diseases, and subjects aged ≥ 65 years regardless of their health conditions, among others [1]. The reasons for protecting the older individuals from influenza include the fact that they have a suboptimal response to the standard-dose influenza vaccine due to immunosenescence and that a large proportion of them (approximately 75% of the population aged 65–75, and more than 85% for those aged > 75) suffer from at least one chronic disease [19]. Moreover, most of the influenza-attributable excess deaths are among individuals ≥ 65 years, and hospitalisation among these subjects often implies suffering from permanent disability afterwards [3, 20].

Influenza and, more generally, flu-like syndromes are difficult to monitor because the associated symptoms are not specific, diagnostic laboratory tests for influenza are not routinely performed and, international diagnosis codes for influenza (International Classification of Diseases, Ninth Revision, Clinical Modification [ICD-9-CM]) are often lacking or incompletely registered in medical records [21]. The combined effect of the sporadic use of laboratory tests for the diagnosis of influenza, the potential occurrence of serious complications, and the difficulties in coding associated hospital events may result in an underestimation of hospital admissions due to influenza and, therefore, its economic impact. Studies assessing the real burden of influenza in Italy are rare, with one provided estimates at the country level [22]. To our knowledge, no studies have measured hospital admissions and relative costs associated with influenza at a regional level in Italy.

## Methods

### Aims and objectives

This study aimed to estimate total hospitalizations and the hospital costs borne by the Italian National Health Service (NHS) associated with influenza at the national and regional levels, from season 2008/09 to 2018/19.

More specifically, the project had the following objectives:

i. To estimate the total number of hospitalizations associated with, including those recorded as such and the estimates of excess hospitalizations associated with influenza, in all Italian regions, that is 19 regions and two Autonomous Provinces (AP) i.e., Bolzano and Trento from the Trentino Alto Adige region, (a total of 21 territorial units, which characterize the Italian territorial organization, hereafter named regions), and by age groups.
ii. To estimate the economic burden (in terms of hospital costs from the Italian healthcare system) of hospitalisations associated with influenza, at the national level.
iii. To estimate the in-hospital mortality associated with influenza.

### Study design

This is a 11-year excess modelling study that used data administrative data collected retrospectively, and included all patients admitted to a hospital for influenza or causes associated with influenza from season 2008/09 to 2018/19, each season going from week 27 of the first year and week 26 of the following year, for a total of 574 weeks.

### Subjects

The study included all patients, of all ages, who had been admitted to any Italian hospital with a valid ICD-CM-9 diagnosis corresponding to influenza or related diagnoses. Hospital admissions were identified based on selected ICD-CM-9 codes (see Table A1) and aggregated for each epidemiological week, region, age groups of 0–4, 5–14, 15–64, and ≥ 65 years, and sex. The choice of age categorisation was dependent on the data structure of influenza incidence (see “Data” section).

### Data

#### Hospital discharge data

Access to Hospital Discharge Records (HDR, Scheda di Dimissione Ospedaliera [SDO]) requires authorisation from the Ministry of Health. Following the introduction of the 2016/679 European Union (EU) Regulation (known as General Data Protection Regulation (GDPR)), the Ministry of Health has adopted a new procedure for the extraction of such data. The authorisation request was submitted to the Ministry of Health on December 2021, requesting the extraction of weekly SDO data for the period 2008–2019, including the information listed in Table A2 of the Additional file 1.

We considered only those hospitalisations with a length of stay > 24 h and primary diagnosis codes related to influenza, based on literature (see for example [21, 23–26]) and further validated by an expert in influenza epidemiology. These comprised: i) influenza (ICD9 code 487): the hospitalizations with primary code 487 are named “observed” throughout the paper, as they represent the known burden of disease; ii) pneumonia other than influenza (ICD9 480–486); iii) respiratory diseases (codes) other than influenza, including 31 respiratory diseases (ICD9 460–466, 480–487, 490–496, 500–508, 510–516, 518); iv) cardiovascular diseases including 17 diagnoses (ICD9 410–414, 422, 427, 428, 430–435, 437, 438, 440); v) other possible associated diagnoses including diagnosis codes related to the nervous system and sense organs (ICD 322, 323, 341, 357, 382); infectious and parasitic diseases (ICD) 040); endocrine, nutritional and metabolic diseases, and immunity disorders (ICD9 250); diseases of the musculoskeletal system and connective tissue (ICD9 728, 729); and congenital anomalies (ICD9 747). All ICD-9-CM codes and corresponding diagnoses are listed in Table A1 of the Additional file 1. We also considered subgroups of these codes related to influenza in a stepwise approach, to enhance comparability with existing published results based on data and techniques similar to those adopted here, in which multiple case definitions and discharge diagnoses have been used to identify potential influenza-associated hospitalizations [27]. We looked first at the codes of pneumonia and influenza (ICD9 480–487), then these codes plus those of the remaining 31 respiratory diseases (ICD9 460–466, 480–487, 490–496, 500–508, 510–516, 518), finally including 17 cardiovascular diseases (ICD9 410–414, 422, 427, 428, 430–435, 437, 438, 440).

For each hospitalization, date of admission and discharge are recorded in the HRD. We, therefore, estimated the average length of stay for admissions with primary code of influenza and other groups of diseases, for each season and for the 11- and latest 3-season average, as shown in the result “Hospitalisations associated with influenza” section.

Hospitalization-related costs are encoded according to the national diagnosis-related group (DRG) ICD-9-CM version for hospitalizations. This system is currently employed in Italy as an instrument for financing the hospital structures in the national health system. Each DRG is associated with a tariff that reflects an estimate of the average cost of each admission, and we used this information to estimate hospitalization costs.

The HDR also contain information on the type of discharge, from which it is possible to know whether the hospitalization ended due to an in-hospital decease.

#### Influenza activity data

The data on the flu incidence at a regional level and by age group were obtained from the national epidemiological and virological surveillance system for influenza (InfluNet), coordinated by the Istituto Superiore di Sanità (ISS), in collaboration with the Interuniversity Center for Influenza Research (CIRI) of Genoa and the support of the Ministry of Health [28]. The incidence measures *influenza-like illness* (ILI) and is expressed as the number of influenza syndromes (cases) per 1,000 patients per epidemiological week. The system of weekly monitoring is based on syndromic surveillance relying on a network of sentinel physicians consisting of General Practitioners (MMG) and primary-care Paediatricians (Pediatri Libera Scelta [PLS]), recruited according to regions, who report cases of influenza syndrome observed among their patients. InfluNet defines an individual with "influenza syndrome" as any person who presents with a sudden and rapid onset of either general symptoms (fever, feverishness, headache, malaise, exhaustion, or muscle aches) or respiratory symptoms (cough, sore throat, or shortness of breath). Data on the incidence of influenza syndrome are available for the period between the 42^nd^ week of a year and the 15^th^/17^th^ week of the following year (except for 2009, in which the survey was conducted throughout the year during the pandemic influenza due to the H1N1 virus). We attributed a value of 0 for the ILI incidence of the missing weeks, following the approach previously adopted in a study using the same data [22].

#### Temperature data

Temperature trends are among the factors that contribute to the spread of the influenza virus [29] and have been considered predictors of hospitalisations associated with influenza in other studies [30]. Data on average temperature were obtained from the National System for the Collection, Processing, and Dissemination of Climate Data of Environmental Interest (Sistema nazionale per la raccolta, l’elaborazione e la diffusione di dati Climatici di Interesse Ambientale [SCIA]) monitoring system coordinated by the Higher Institute for Environmental Protection and Research (Istituto Superiore per la Protezione e Ricerca Ambientale [ISPRA]) [31]. The national and regional weekly average temperatures were estimated as the average of temperatures measured by about 1,000 weather stations belonging to the monitoring system network.

#### Demographic data

Finally, to compute the rates as cases per 100,000 people, data on age-specific seasonal resident population estimates were downloaded from the Institute of National Statistics (ISTAT)’s website [32].

### Outcomes

According to the research objectives, the outcomes of the study are the following:

- *Total number of hospitalizations associated with influenza:* this is given by the sum of “observed” hospitalizations due to influenza, i.e., those recorded with primary ICD9 code 487, and “excess” hospitalizations, that are those estimated by the model described in “Data analysis” section, that are hospitalizations recorded with primary ICD9 codes different from 487, but considered associated with influenza based on the model assumptions.
- *Cost of hospitalisations associated with influenza:* this is an approximated estimate since it refers to excess (i.e., estimated) rather than observed hospitalisations. Hence, we hypothesised a range of possible attributable costs, ranging from prudential estimates, i.e., attributing to hospitalizations associated with influenza the same cost of hospitalizations coded with primary diagnosis influenza, to wide scope-estimates considered more realistic, where the average cost of hospitalizations for cardio-respiratory diagnoses is attributed to hospitalizations associated with influenza. See “Economic burden of hospitalisations associated with influenza” section.
- *Total in-hospital mortality associated with influenza:* this is given by the sum of “observed” in-hospital deaths for hospitalizations due to influenza, i.e., those deaths recorded in hospitalizations with primary ICD9 code 487, and in-hospital deaths attributable to influenza, that are those estimated by the model described in “Data analysis” section, that come from hospitalizations recorded with primary ICD9 codes different from 487, but considered associated with influenza based on the model assumptions.

### Data analysis

#### Hospitalisations associated with influenza

For the hospitalisation model, we relied on the approach developed in existing literature applying regression modelling techniques to estimate influenza-associated hospitalizations and mortality. Such approach was first developed for estimating influenza-associated mortality in the United States (US) [33] and has been widely applied ever since [24, 34, 35], then modified to estimate the numbers and rates of influenza-associated hospitalisations [23]. The most common regression techniques used for analysing the count of rare, repeatable and independent events are Poisson and negative binomial models [27]. Here we used an age-specific negative binomial regression model with a log link. At the national level, the age-specific negative binomial regression models that we used can be written as

1 $$Log(E(Y))= {\upbeta }_{0}+{\upbeta }_{1}[{\text{t}}]+{\upbeta }_{2}[\mathrm{sin }(2\mathrm{t\pi }/52.18)]+{\upbeta }_{3}[{\text{cos}}(2\mathrm{t\pi }/52.18)]+{\upbeta }_{4}[{\text{I}}]+{\upbeta }_{5}[{\text{T}}]$$

where *Y* represents the number of hospitalisations during a particular week for the group of diagnoses associated with influenza. Specifically, hospital admissions with ICD-9-CM diagnosis code of influenza (code 487) were excluded from the model (parameter Y), as such hospitalisations are assumed to be associated with influenza, and thus, should not be used for modelling [22, 23]. *t* is the number of weeks in a time series from October 2008 (week 27, numbered 1) to April 2019 (week 26, numbered 574); *I* is the incidence of influenza syndrome expressed as the number of cases per 1000 patients per week; and *T* is the average weekly temperature. The estimated coefficients are as follows: β_0_ is the intercept; β_1_ accounts for linear time trends (in weeks); β_2_ and β_3_ account for the seasonality of hospitalisations; and β_4_ and β_5_ are coefficients associated with the incidence of influenza and temperature, respectively (coefficient estimates of the negative binomial regression for all ICD9 codes considered, all ages, at national level are available in Table A3 of the Additional file 1).

The model fitting included testing the various lags and moving averages of influenza indicators to account for delays between disease onset and hospitalisation. We selected the best model based on the Akaike Information Criterion (AIC) [34] (see Additional file 1 Table A4) estimated for the models considering all-age hospitalisations for all selected ICD9 codes associated with influenza, at the national level.

Thereafter, we applied the selected negative binomial model to the age-specific (0–4, 5–14, 15–64, and ≥ 65 years) and region-specific time series (i.e., considering age-specific and region-specific hospitalizations and ILI incidence time series). Furthermore, the models were replicated considering as *Y* various diagnosis subgroups of hospitalisations based on the ICD-9-CM code described above (not including ICD9 code 487), specifically respiratory (ICD9 460–466, 480–486, 490–496, 500–508, 510–516, 518), and cardiorespiratory diagnosis groups (ICD9 for respiratory diseases plus ICD9 410–414, 422, 427, 428, 430–435, 437, 438, 440).

Outcome measures obtained from the model include numbers, representing the number of excess hospitalizations associated with influenza, and rates, expressed per 100,000 population, where population is the age- and region-specific population in each season. Specifically, to estimate the number of hospitalisations not directly associated with influenza, the hospitalisations predicted using the model(s) just described were subtracted from those estimated with the same model in which the variable relating to the incidence of the influenza syndrome was set to 0 (baseline). In this way, an attempt was made to separate hospitalisations for respiratory, circulatory and other pathologies from those related to patients not infected by the influenza virus [22, 33].

Finally, as a sensitivity analysis, we replicated the above model by additionally including admissions with ICD-9-CM primary diagnosis code of influenza.

#### Economic burden of hospitalisations associated with influenza

To assess costs related to influenza-attributed hospital admissions, we approximated their estimate since we deal with estimated rather than observed hospitalisations.^1^ We estimated a range of costs for hospitalisations associated with influenza, going from conservative to more wide scope estimates. Prudential estimates were meant as the minimum attributable cost and estimated applying the mean hospitalisation cost for influenza (ICD-9-CM code 487) to the estimated number of influenza-associated excess hospitalisations. Then, a wide-scope value was estimated by applying the mean hospitalisation cost for cardiorespiratory diseases. With either assumption, the total cost of hospitalisations for influenza was defined as the sum of the cost of observed hospitalisations with a primary diagnosis of influenza and the cost of excess hospitalisations associated with influenza, for which we considered the two approximations just described, using the mean hospitalization cost for influenza, likely to underestimate the real economic burden of hospitalizations, and a wide scope estimate, considering mean hospitalization cost for cardiorespiratory hospitalizations.

#### In-hospital mortality associated with influenza

Influenza-associated death have mostly been studied in literature based on national mortality data, which refers to the overall mortality associated with influenza rather than the in-hospital mortality. One study [36] considered in-hospital mortality as a type of influenza-associated critical illness hospitalisation and provided an estimate by re-adapting the abovementioned method.

In our study, we estimated Eq. (1) with Y representing the number of in-hospital deaths recorded for admissions with primary codes of influenza and influenza-related diseases as listed in Table A1. All other parameters are the same as described above. Consistently, to estimate the number of in-hospital deaths not directly associated with admissions due to influenza, the deaths predicted using the model just described were subtracted from those estimated with the same model in which the variable relating to the incidence of the influenza syndrome was set to 0 (baseline).

As for excess hospitalizations associated with influenza, outcome measures of excess in-hospital mortality associated with influenza include numbers, representing the number of in-hospital deaths associated with influenza, and rates, expressed per 100,000 population.

All statistical analyses were performed using Stata 17 software (StataCorp LP, College Station, TX, 2015).

### Patient and public involvement

Patients or the public were not involved in the design, conduct, reporting, or dissemination of our research.

## Results

### Hospitalisations associated with influenza

#### Observed hospitalizations for influenza

From the 2008/09 to 2018/19 seasons, the mean incidence of ILI was 3.5 per 1000 patients, with the highest incidence (4.4) observed in the latest two seasons. ILI incidence was the highest among the youngest population. Particularly, ILI incidence was much higher in children aged 0–4 years than in the total population, from 2.4 to 2.7 times higher throughout the observation period (Table 1).

**Table 1:** Mean seasonal incidence of ILI per 1000 patients, seasons 2008/09 – 2018/19, Italy

Season^a^	Total	0–4 years	5–14 years	15–64 years	≥ 65 years
2008/09	2.5	6.6	4.8	2.0	1.2
2009/10	3.3	8.1	9.2	2.3	0.9
2010/11	3.5	9.1	7.00	2.9	1.0
2011/12	3.0	7.9	4.5	2.6	1.3
2012/13	3.6	9.1	6.7	3.2	1.3
2013/14	2.7	7.4	4.2	2.5	1.1
2014/15	3.7	8.9	6.0	3.5	1.6
2015/16	2.9	7.9	5.7	2.4	1.0
2016/17	3.2	8.0	4.6	3.0	1.8
2017/18	5.1	13.5	7.4	4.7	2.6
2018/19	4.8	13.1	6.9	4.5	2.2
*11-season mean*	*3.5*	*9.0*	*6.1*	*3.0*	*1.5*
*3-season mean*^b^	*4.4*	*11.5*	*6.3*	*4.07*	*2.2*

*ILI* Influenza-like illness

^a^Week 42 to week 17 of the following year are covered

^b^Includes the most recent seasons, namely 2016/17, 2017/18, and 2018/19

**Fig. 1:**
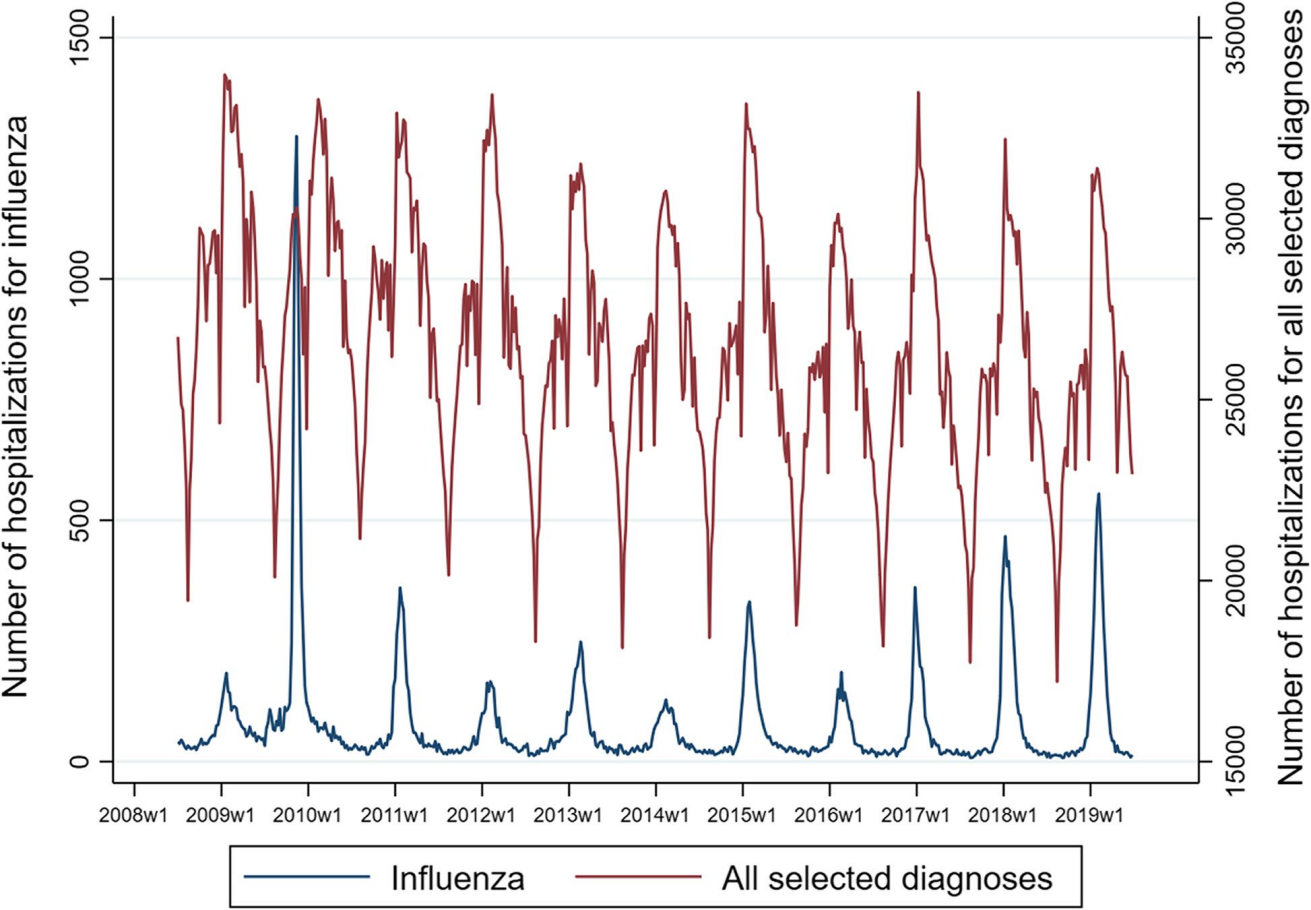
Weekly numbers of hospitalisations by primary discharge type, seasons 2008/09 – 2018/19, Italy, all ages. Note: w = week

During the observation period, the average number of hospitalisations with a primary diagnosis of influenza was 3,969 (sd, 1,853) per season (Table 2). The average seasonal number of hospitalisations with either primary or secondary diagnosis of influenza was 7,571 (data presented Table A5 in the Additional file 1). The average number of hospitalizations per season with a primary diagnosis of pneumonia and influenza was 140,646; of respiratory system diseases was 483,834; and of cardiorespiratory diseases was 1,319,496, based on the selected ICD9 codes (Table A5 in the Additional file 1). All groups of diagnoses had a seasonal trend over the observation period, with hospitalisations for influenza showing, overall, a slightly increasing trend between 2008/09 and 2018/19 and those for all selected diseases associated with influenza presenting an overall slightly declining trend (Fig. 1).

**Table 2:** Observed hospitalisations with primary diagnoses of influenza and ICD9 codes associated with influenza, by age groups. Seasons 2008/09 – 2018/19, Italy

Season^a^		Influenza (ICD9 diagnosis 487)					All ICD9 codes associated with influenza^b^				
		0–4 years	5–14 years	15–64 years	≥ 65 years	Total	0–4 years	5–14 years	15–64 years	≥ 65 years	Total
2008/09	N	1,095	463	1,028	815	3,404	72,886	20,882	340,084	1,048,895	1,483,008
	Rate^c^	39.0	8.4	2.6	6.8	5.7	2,595.9	377.5	874.0	8,769.0	2,504.6
2009/10	N	2,468	1,565	4,092	918	9,054	71,147	23,945	347,086	1,053,095	1,495,772
	Rate	87.5	28.1	10.5	7.6	15.2	2,521.7	429.7	888.4	8,708.3	2,511.6
2010/11	N	1,163	577	1,523	759	4,022	67,592	22,396	335,004	1,026,908	1,451,913
	Rate	41.3	10.3	3.9	6.2	6.7	2,398.2	399.3	854.9	8,414.4	2,427.2
2011/12	N	906	277	742	706	2,631	59,412	17,114	313,565	1,020,906	1,411,006
	Rate	32.4	4.9	1.9	5.7	4.4	2,122.4	303.6	799.9	8,241.8	2,350.6
2012/13	N	1,189	442	1,058	760	3,449	57,617	17,577	307,113	992,889	1,375,196
	Rate	42.9	7.8	2.7	6.0	5.7	2,078.0	310.4	784.9	7,861.1	2,284.7
2013/14	N	970	343	638	496	2,447	54,658	19,873	297,979	988,950	1,361,461
	Rate	35.5	6.0	1.6	3.9	4.1	1,998.7	349.8	763.3	7,690.0	2,257.4
2014/15	N	1,171	408	1,162	1,066	3,807	55,362	18,429	298,718	1,004,708	1,377,221
	Rate	43.8	7.2	3.0	8.1	6.3	2,070.6	324.1	768.3	7,681.0	2,283.2
2015/16	N	830	372	680	600	2,482	52,595	16,794	291,492	991,361	1,352,242
	Rate	31.9	6.6	1.8	4.5	4.1	2,021.5	296.1	753.1	7,481.1	2,245.1
2016/17	N	756	290	768	1,314	3,128	52,248	15,683	283,149	986,112	1,337,192
	Rate	29.9	5.1	2.0	9.8	5.2	2,067.2	278.0	734.4	7,363.7	2,224.4
2017/18	N	1,331	447	1,196	1,622	4,596	49,981	14,732	284,626	971,698	1,321,038
	Rate	54.3	8.0	3.1	12.0	7.7	2,037.7	262.8	740.6	7,190.5	2,201.6
2018/19	N	1,115	355	1,331	1,841	4,642	50,224	17,164	284,540	968,675	1,320,630
	Rate	46.9	6.4	3.5	13.5	7.8	2,113.3	308.7	742.7	7,107.4	2,205.6
Total	N	12,994	5,539	14,218	10,897	43,662	643,722	204,589	3,383,356	11,054,197	15,286,679
*11-season mean*	N	*1,181.3*	*503.5*	*1,292.5*	*990.6*	3,969.3	*58,520.2*	*18,599.0*	*307,577.8*	*1,004,927.0*	1,389,698.1
	Rate	*44.2*	*9.0*	*3.3*	*7.7*	*6.6*	*2,190.5*	*330.7*	*791.6*	*7,839.7*	*2,317.4*
*3-season mean*^d^	N	*1,067.3*	*364.0*	*1,098.3*	*1,592.3*	*4,122.0*	*50,817.7*	*15,859.7*	*284,105.0*	*975,495.0*	*1,326,286.7*
	*Rate*	*40.0*	*6.5*	*2.8*	*12.4*	*6.9*	*1,902.2*	*282.0*	*731.2*	*7,610.1*	*2,211.6*

^a^Each season extends from week 27 of one year to week 26 of the following year

^b^See list of ICD9 codes in Table A1 of the Additional file 1

^c^Every 100,000 inhabitants

^d^Includes the most recent seasons, namely 2016/17, 2017/18, and 2018/19

Numbers stratified by age groups indicated that the highest numbers and rates of hospitalisations with primary code influenza were recorded among the paediatric population aged 0–4 years, whilst hospitalisations for all ICD9 codes associated with influenza were highest among individuals ≥ 65 years. Age-specific numbers per respiratory and cardiorespiratory hospitalisations are presented in Additional file 1 Table A6.

#### Excess hospitalisations associated with influenza

Table 3 presents estimates of influenza-associated hospitalisations by age group. Coefficient estimates, for all ages, are available in Additional file 1 Table A3.

**Table 3:** Estimated numbers and rates per 100,000 people of excess hospitalization associated with influenza, by age group. Seasons 2008/09 – 2018/19, Italy

Season^a^	0–4 years		5–14 years		15–64 years		≥ 65 years		Total	
	N	Rate^b^	N	Rate^b^	N	Rate^b^	N	Rate^b^	N	Rate^b^
2008/09	2207	78.6	666	12.0	3171	8.2	13971	116.8	16,503	27.9
2009/10	1932	68.5	1193	21.4	3203	8.2	10042	83.0	20,613	34.8
2010/11	2749	97.5	941	16.8	4275	10.9	10895	89.3	22,797	38.5
2011/12	2315	82.7	585	10.4	3771	9.6	14937	120.6	18,966	32.0
2012/13	2535	91.4	851	15.0	4536	11.6	14791	117.1	23,241	39.3
2013/14	1871	68.4	480	8.4	3416	8.8	12418	96.6	16,849	28.5
2014/15	2197	82.2	696	12.2	4759	12.2	18070	138.1	22,885	38.7
2015/16	1788	68.7	632	11.1	3212	8.3	10453	78.9	17,203	29.1
2016/17	1756	69.5	467	8.3	3950	10.2	19295	144.1	19,073	32.2
2017/18	2988	121.8	763	13.6	6069	15.8	28959	214.3	30,556	51.6
2018/19	2767.7	116.5	701	12.6	5679	14.8	23542	172.7	28,211	47.6
*Total*	*25,106*		*7,975*		*46,042*		*177,373*		*236,896*	
*11-season mean*	2282	86.0	725	12.9	4186	10.8	16125	124.7	21536	36.4
3-season mean^c^	2503.9	102.6	643.7	11.5	5232.7	13.6	23932	177.0	25946.7	43.8

^a^Each season extends from week 27 of one year to week 26 of the following year

^b^Every 100,000 inhabitants

^c^Includes the most recent seasons, namely 2016/17, 2017/18, and 2018/19

In the period between seasons 2008/09 and 2018/19, approximately 44,000 hospitalisations with a primary diagnosis of influenza were observed and more than 15 million hospitalisations had a primary diagnosis associated with influenza (columns “Total” of Table 2). Over the same period, the excess hospitalization associated with influenza was estimated to be more than five times higher, corresponding to 236,896 hospitalisations when considering all diagnoses associated with influenza (Table 3), indicating that a share of hospitalizations with a primary diagnosis different from influenza were, in fact, to be attributed to influenza. Furthermore, the number of excess hospitalization was similar for respiratory and cardiorespiratory subgroups of codes (see Additional file 1 Table A7). Overall, numbers and rates per 100,000 people of excess hospitalization associated with influenza increased over time. For example, estimated rates increased from 27.9 in season 2008/09 to 47.6 in season 2018/19 (last column of Table 3), while observed rates of influenza hospitalisations increased from 5.7 to 7.8 (column “Total” for Influenza (ICD9 diagnosis 487) in Table 2).

Results were replicated including also hospitalisations with diagnosis ICD9 code 487. Estimates were higher regarding the number of observed hospitalisations for influenza (Table A8).

Looking at results by age group, we observed that the highest excess hospitalisation associated with influenza occurred among individuals ≥ 65 years, although the incidence of influenza was the lowest in this group (Table 1). Compared to the observed number of hospitalisations with a primary diagnosis of influenza (see Table 2) that were highest among the 0–4-year age group, the excess numbers and rates of hospitalization associated with influenza were considerably concentrated in the age group of ≥ 65 years. Results for excess hospitalization associated to influenza for respiratory and cardiorespiratory diagnoses, by age, are presented in Table A9 of the Additional file 1.

**Fig. 2:**
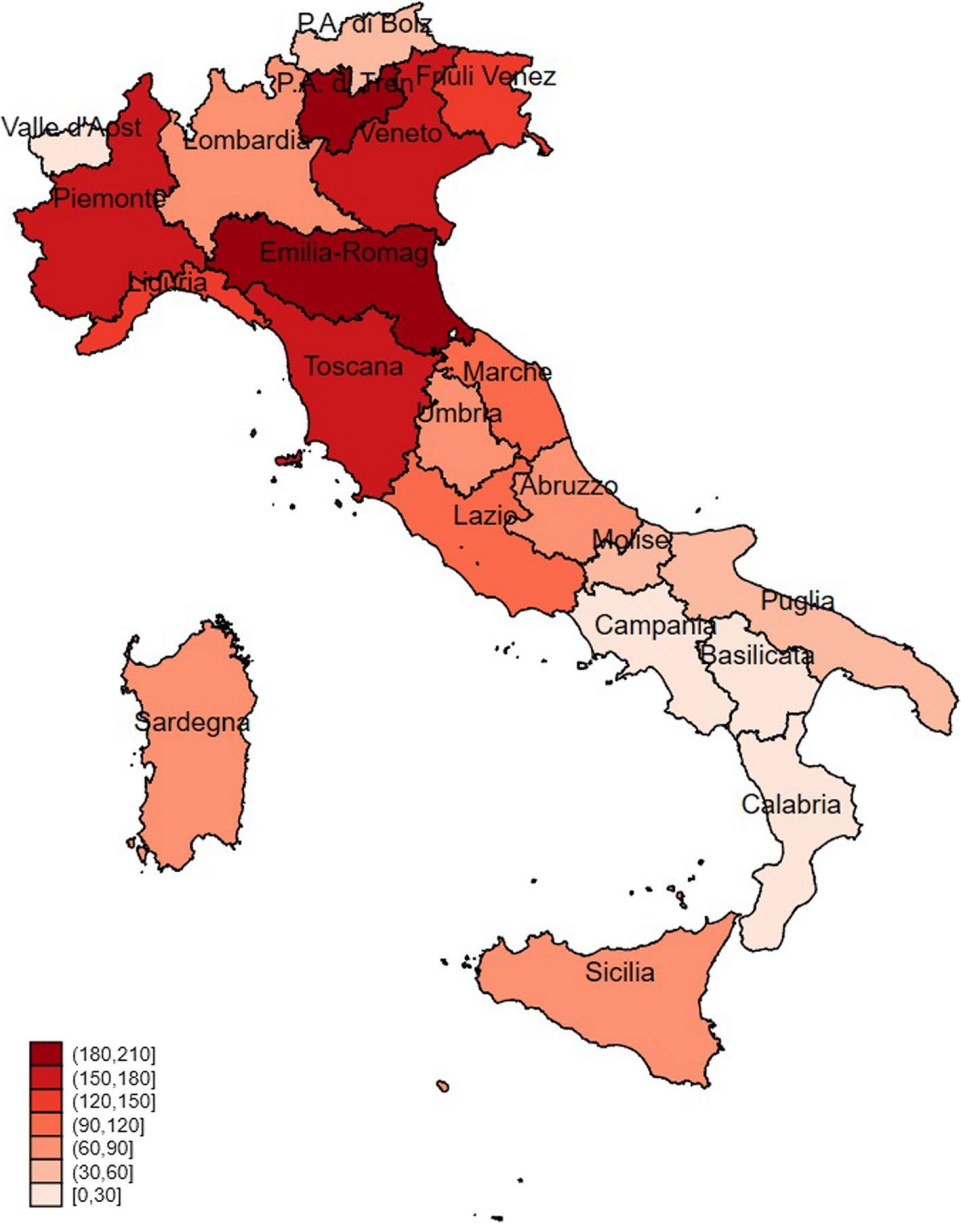
Average rates per 100,000 people of excess hospitalization associated with influenza from seasons 2008/09 to 2018/19 among the population aged ≥ 65 years, by region in Italy. Note: Valle d’Aosta, Basilicata, and Calabria had data on age-specific incidence with several missing values; hence, estimates are unreliable

The analyses were replicated following categorisation by region (descriptive results on observed hospitalization with primary diagnosis of influenza are available in Table A10 and analytical results on estimates of excess hospitalizations associated with influenza are available in Table A11 of the Additional file 1). To provide comparative evidence between regions at glance, we focused on population aged 65 -individuals at highest risk of hospitalizations, to compare more homogeneous groups and avoid comparisons between regions with different age structures. Figure 2 reports the 11-season mean rates of excess influenza-associated hospitalisations for each Italian region among the population aged ≥ 65 years (regional results for the total population are available in Figure A1 of the Additional file 1). The results reveal heterogeneity across geographical regions in terms of excess hospitalizations associated with influenza per 100,000 people. This is partly interpretable as a geographical gradient, where most but not all northern regions tend to have higher rates. Furthermore, when interpreting results, it must be accounted that age-specific numbers (both hospitalisations and ILI rates) for small regions presented numerous missing values making estimates for this group unreliable. To put our results into perspective and further interpret them, Tables A12 and A13 of the Additional file 1 report the average seasonal ILI rate per region for individuals ≥ 65 years and the numbers of hospitalisations with primary code 487 per 100,000 people by region for the same population, respectively. Among people aged 65 + , regions with the highest rates of excess influenza-associated hospitalisations (see Fig. 2) tend to be also those with the highest registered rates of hospitalisations for influenza (see Table A13).

### Economic burden of hospitalisations associated with influenza

Herein, we present the length of stay and hospitalisation costs for admissions with primary code of influenza and cardiorespiratory diseases, given that the estimated costs associated with influenza are based on these two sets of hospitalisations. The average length of stay due to influenza over the observation period was 5.5 days. It increased over time from 4.9 days in 2008/09 to 6.7 in 2018/19. The average length of stay for cardiorespiratory conditions remained constant over seasons and was almost twice as much as that of admissions with a diagnosis of influenza at each season, with a mean value of 10.4 days between 2008/09 and 2018/19 (Table 4). The average hospitalisation cost, based on DRG, was €2159 for influenza and €5192 for cardiorespiratory diagnoses, which is more than twice as that for influenza.

**Table 4:** Average length of stay and cost of hospitalisation with primary diagnoses of influenza and cardiorespiratory diagnoses associated with influenza. Seasons 2008/09 – 2018/19, Italy

Season^a^	Influenza (ICD9 code 487, primary)		Cardiorespiratory diseases^c^	
	Average length of stay (days)	Average cost (€)	Average length of stay (days)	Average cost (€)
2008/09	4.9	1581.1	10.3	4093.4
2009/10	5.1	2224.9	10.3	4979.8
2010/11	5.2	2221.4	10.3	5091.0
2011/12	5.1	1646.2	10.3	5166.1
2012/13	5.2	1985.5	10.3	5211.5
2013/14	4.7	1822.9	10.3	5258.0
2014/15	5.5	2822.4	10.4	5350.5
2015/16	5.6	2214.6	10.5	5444.7
2016/17	5.8	1956.6	10.4	5463.4
2017/18	6.1	2776.1	10.5	5497.5
2018/19	6.7	2496.9	10.5	5560.8
*11-season mean*	*5.5*	*2159.0*	*10.4*	*5192.4*
*3-season mean*^b^	*6.2*	*2409.9*	*11.1*	*4856.9*

^a^Each season extends from week 27 of one year to week 26 of the following year

^b^Includes the most recent seasons, namely 2016/17, 2017/18, and 2018/19

^c^Include ICD9 codes for influenza (ICD9 code 487); ii) pneumonia other than influenza (ICD9 480–486); iii) respiratory diseases (codes) other than influenza (ICD9 460–466, 480–487, 490–496, 500–508, 510–516, 518); iv) cardiovascular diseases (ICD9 410–414, 422, 427, 428, 430–435, 437, 438, 440)

**Fig. 3:**
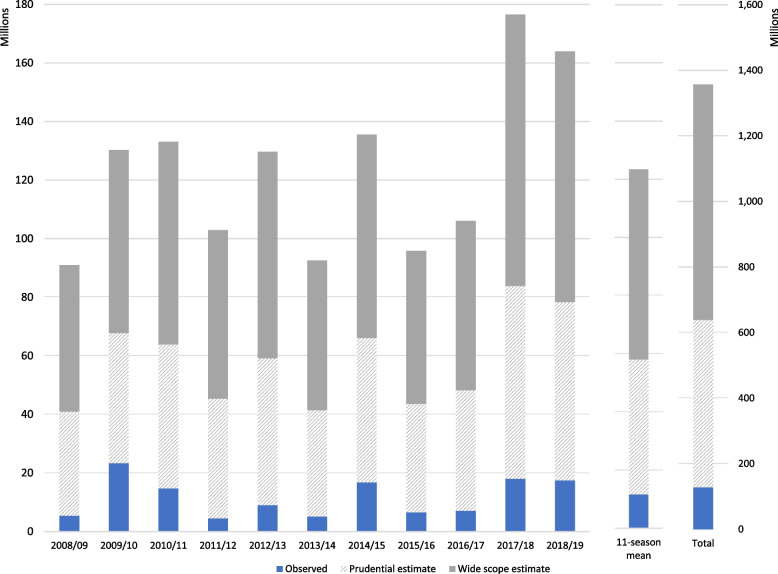
Seasonal costs of influenza and excess hospitalisations associated with influenza. Seasons 2008/09 – 2018/19, Italy

The observed average cost of hospitalizations with primary diagnosis influenza was on average €11,548,296 per season (Fig. 3 and Table A14 of the Additional file 1). To estimate the cost of the additional excess hospitalisations associated with influenza, we approximated its value with the average cost of hospitalisation with a primary diagnosis of cardiorespiratory diseases between seasons 2008/09 and 2018/19 (€5,192) while considering a ‘lower bound value’, which represents a more prudent estimate, based on the average cost of hospitalisations with a primary diagnosis of influenza (€ 2,159). The prudential estimate is considered highly conservative since the estimated additional costs should reflect the cost of hospitalisations according to the type of hospitalizations associated with influenza (which is mainly for diagnosis of cardiorespiratory diseases). Calculated thus, the average seasonal cost of the additional excess hospitalisations was €111,824,361 (prudential estimate, €46,495,382) per season and the total hospital expenditure over the 11 seasons summed up to €123,372,657 (prudential estimate, €58,043,678) (Table A14).

### In-hospital mortality associated with influenza

The Hospital Discharge Records do not provide information on the cause of the deaths that occurred in the hospital. Nevertheless, in-hospital mortality related to admissions with a primary diagnosis of influenza or influenza-associated diseases may provide some insight into mortality associated with influenza. Herein, we offer a descriptive and analytical overview of this matter. Table 6 presents in-hospital mortality by age group and season for admissions with a primary diagnosis of influenza and for hospitalisations associated with influenza. Most deaths occurred in the oldest age group, and the group least affected was children aged 5–14 years.

Table 6 presents the excess in-hospital mortality associated with influenza (observed number of in-hospital mortality by different groups of diagnoses at hospital admission are shown in table A15). Results are also available by region (Tables A16 and A17 of the Additional file 1). Compared to in-hospital deaths observed for admissions with a primary diagnosis of influenza (second to sixth columns of Table 5), the number of excess in-hospital deaths associated with influenza were much higher in the age group ≥ 65 years (Table 6). According to the model, approximately 5% of the deaths registered in hospitals for all diagnoses considered were to be associated with influenza (910,864 vs 39,941 in the total population (see row “Total” Table 5 column “All ICD9 associated with influenza – Total” and Table 6 column “Total”, respectively) and 834,138 vs 45,199 in the population aged ≥ 65 years (see row “Total” Table 5 column “All ICD9 associated with influenza – ≥ 65 years” and Table 6 column “ ≥ 65 years”, respectively)).

**Table 5:** Observed in-hospital mortality for admissions with primary diagnoses of influenza and ICD9 codes associated with influenza (numbers), by age group and season

Season^a^	Influenza (ICD9 diagnosis 487)					All ICD9 associated with influenza				
	0–4 years	5–14 years	15–64 years	≥ 65 years	Total	0–4 years	5–14 years	15–64 years	≥ 65 years	Total
2008/09	0	0	1	14	15	153	61	6,674	71,286	78,184
2009/10	0	3	48	25	76	125	57	6,896	72,227	79,319
2010/11	0	0	44	19	63	130	68	7,057	72,410	79,666
2011/12	0	0	3	13	16	127	52	6,756	75,940	82,875
2012/13	0	0	8	15	23	113	69	6,792	72,943	79,917
2013/14	0	0	2	5	7	120	64	6,408	72,655	79,247
2014/15	0	0	34	55	89	112	61	7,000	79,204	86,377
2015/16	0	0	12	13	25	114	71	6,720	77,371	84,276
2016/17	0	0	8	49	57	114	59	6,790	81,387	88,350
2017/18	0	1	17	47	65	127	81	6,845	78,976	86,029
2018/19	1	0	22	94	117	105	60	6,720	79,739	86,624
*Total*	*1*	*4*	*199*	*349*	553	*1,339*	*703*	*74,658*	*834,138*	910,864
11-season mean	0	0.4	17.7	25.5	50.3	123.5	64.3	6,793.8	75,439.9	82,805.8
3-season mean^b^	0.3	0.3	15.7	63.3	79.7	115.3	66.7	6785	80034	87001

^a^Each season extends from week 27 of one year to week 26 of the following year

^b^Includes the most recent seasons, namely 2016/17, 2017/18, and 2018/19

**Table 6:** Estimates of numbers and rates per 100,000 people of excess in-hospital mortality associated with influenza, by age group and season

Season^a^	0–4 years		5–14 years		15–64 years		≥ 65 years		Total	
	N	Rate^b^	N	Rate	N	Rate	N	Rate	N	Rate
2008/09	4	0.1	3	0.0	253	0.6	3157	26.4	2,424	4.1
2009/10	4	0.1	6	0.1	267	0.7	2271	18.8	3,196	5.4
2010/11	5	0.2	4	0.1	358	0.9	2517	20.6	3,553	5.9
2011/12	4	0.1	3	0.0	319	0.8	3555	28.7	2,985	5.0
2012/13	4	0.2	4	0.1	393	1.0	3568	28.2	3,764	6.3
2013/14	3	0.1	2	0.0	297	0.8	3045	23.7	2,771	4.6
2014/15	4	0.2	4	0.1	432	1.1	4594	35.1	3,900	6.5
2015/16	3	0.1	4	0.1	286	0.7	2614	19.7	2,941	4.9
2016/17	3	0.1	3	0.1	372	1.0	5214	38.9	3,393	5.6
2017/18	69	0.2	5	0.1	602	1.6	8176	60.5	5,700	9.5
2018/19	6	0.2	5	0.1	563	1.5	6489	47.6	5,313	8.9
*Total*	*46*		*42*		*4,141*		*45,199*		*39,941*	
11-season mean	4	0.2	4	0.1	377	1.0	4,109	31.7	3,631	6.1
3-season mean^c^	26	0.2	4.3	0.1	512.3	1.4	6626.3	49	4802	8

^a^Each season extends from week 27 of one year to week 26 of the following year

^b^Every 100,000 inhabitants

^c^Includes the most recent seasons, namely 2016/17, 2017/18, and 2018/19

## Discussion

According to InfluNet, influenza-like syndromes affect 9% of the Italian population each year, on average, with a minimum of 4% observed in the 2005–06 season and a maximum of 15% recorded in the 2017–18 season [37]. The Italian study of Rosano et al. [3] found that over 68,000 deaths had to be attributed to influenza epidemics between seasons 2013/14 and 2016/17, with the annual mortality excess rate per 100,000 ranging from 11.6 to 41.2. Most influenza-associated deaths per year were registered among the older population [3]. In the 2014/15 and 2016/17 seasons, higher influenza-attributable excess death rates were also observed among children < 5 years [3]. A recent systematic review on the burden of seasonal influenza in Italy identified 16 studies covering different settings and periods, and the evidence suggests that, each year, influenza places a significant burden especially on high-risk groups in terms of complications, hospitalisations, and mortality [38].

Using hospital discharge records combined with data on ILI incidence, we estimated the excess hospitalization associated with influenza and related economic burden in the period between 2008/09 and 2018/19. We built on previous evidence produced for Italy by Bertolani et al. [22] and extended the observation period up to pre-pandemic season 2018/19. Furthermore, we assessed the burden of influenza among different age groups and regions and investigated excess in-hospital mortality related to influenza. Over the observation period, an average of approximately 21,500 excess hospitalisations were attributed to the influenza virus per season, corresponding to 36.4 cases per 100,000. Additionally to these, the observed hospitalisations with a confirmed diagnosis of influenza were, on average, 3,970 per season. When disentangling results by age groups (hence using age-specific ILI incidence instead of population average and hospitalisations by age group such that the sum of age-specific analyses does not coincide with population estimates), most of the excess hospitalisation associated with influenza pertained to older adults (an average of approximately 16,000). The highest rates were recorded among older individuals (aged 65 +), followed by paediatric patients aged 0–4 years, with 125 and 86 hospitalisations per 100,000 inhabitants, respectively. To put these findings in perspective, recent evidence produced in Spain and Portugal in similar seasons also suggested a relevant under-detected burden of influenza, mostly in the older population. In Spain, the mean annual influenza-associated hospitalisations were estimated at 34,894, corresponding to 75.0 cases per 100,000, while in Portugal, estimates were 5,356 hospitalisations and 51.5 cases per 100,000. Numbers for the population aged ≥ 65 years rose to 335.3 and 200 cases per 100,000, respectively, in Spain and Portugal. The mean direct annual cost of the estimated excess hospitalisations was €142.9 million in Spain and 15.2 million in Portugal, where the estimated excess hospitalisations associated with influenza were one-seventh of those in Spain [25, 26].

The fact that the burden of influenza, in terms of hospitalisations and in-hospital mortality, is mostly borne by older adults, despite the ILI incidence being higher in young children has important implications. As the pandemic emphasised, children often act as a vehicle in the diffusion of the contagion, and therefore preventive measures, such as vaccine campaigns, should be directed at all subjects. An important aspect to consider is that influenza surveillance in Italy is syndromic and not virologic. The ILI incidence, therefore, does not refer exclusively to influenza, but to other viruses too, hence overestimating the circulation of influenza. In contrast, laboratory-confirmed virus infection is limited and underestimates the phenomenon. Therefore, the overall burden cannot be exclusively attributed to the influenza virus and includes other viruses as well, and this may have biased the analysis’s results. This data limitation indeed emerged even more clearly during the post-pandemic phase. The latest report on virological surveillance by InfluNet, covering from week 46 of 2022 to week 9 of 2023, reveals that, out of 21,933 clinical samples collected by the various laboratories (not representative at the national level), 25% tested positive for the influenza virus (88.6% type A and 11.4% type B); 7% for SARS-CoV-2; and 22% for other respiratory viruses, mostly RSV (14%) [39]. This highlights that ILI, as captured by the syndromic surveillance, includes not only influence but other respiratory viruses, and this should be considered when linking ILI to influenza hospitalisations.

Excess hospitalization rates associated with influenza varied drastically across regions. While this represents a novel contribution of this study, it also opens up further investigations to understand the reasons for such variations when considering the older population (≥ 65 years) and the total regional population. While a geographical gradient was expected, heterogeneity within macro-regions was also noted: for example, Lombardy presented lower rates of hospitalisations with a primary diagnosis influenza as well as lower absolute numbers of estimated excess hospitalisations associated with influenza than Emilia-Romagna. The comparison of these two regions is particularly challenging, considering that Emilia Romagna has higher vaccine coverage [40]. Differences in coding hospitalisations or ILI reporting may contribute to explain this observation. However, to date, these are hypotheses which have not been tested and should be further investigated.

The systematic review on the burden of influenza in Italy, published in 2022 [38], found that in the general population, complications due to influenza occurred in 35% of patients visited by general practitioners (GPs), and older patients and those with concomitant chronic diseases were at higher risk. Respiratory complications were most frequently studied, with bronchitis and pneumonia accounting for 43.2% of the complications. Additionally, non-respiratory complications, such as cardiac and neurological issues, were reported mostly in the adult population, while acute otitis media was mostly reported in children. The diagnoses of hospitalisations associated with influenza considered in this study are in line with those commonly used in literature [41] and are coherent with findings reported in the systematic review, highlighting the fact that the estimated burden of influenza on hospitalisations measured using the ICD-9-CM code 487 alone do not completely capture the effect of influenza virus activity. While this conclusion is common to all studies, it is possible for the modelled rates of hospitalization rates and outcomes attributed to influenza to be overestimated to different extents across research. In particular, the availability of data on laboratory-based surveillance for influenza viruses -where laboratories provide numbers of total respiratory specimens tested for influenza and positive influenza tests by virus type and subtype, may limit the risk of overestimation and make estimates more precise and therefore informative in the field and to policy-making.

We also estimated excess in-hospital mortality associated with influenza. To the best of our knowledge, this has never been investigated in Italy in the general population. While we are limited in the analysis because the cause of in-hospital death is unknown from HDR, we believe the results of the analysis provide new insights into the interpretation of available estimates on excess mortality due to influenza. A recent study of the Italian, Portuguese and Cypriot oldest-old patients (≥ 85 years old) reported that death occurred in 13.9% of hospitalised patients with laboratory-confirmed influenza and/or respiratory syncytial virus infection or that developed it during the course of admission for other causes [42]. We found that the average seasonal number of in-hospital deaths for admissions with a primary or secondary diagnosis of influenza was approximately 150, while observed in-hospital deaths occurring for admissions with a primary diagnosis of pneumonia and influenza was approximately 9,500 (see Table A15 in the Additional file 1). The estimated in-hospital mortality attributable to with influenza indicates that, on average, as many as 3,600 extra in-hospital deaths might be attributed to influenza virus circulation. In the case of mortality, numbers are to be almost exclusively attributed to the older population. Regional variations were quite remarkable, with rates ranging from 3.8 to 11, partly depending on regional population age structure; however, further research is needed to investigate regional inequalities arising from differences in the regional healthcare systems.

Also, despite providing a broader perspective on the cost of treating severe influenza, the study still shows only a fraction of the economic burden. The burden of milder influenza cases with self-management, or those cases managed in primary health care setting were not included. Indirect costs of lost productivity (from patients or their caregivers) and the costs related to the long-term care caused or aggravated by influenza were not computed.

## Conclusions

Our analysis refers to pre-pandemic times, and we believe this strengthens the validity of our results because just before and during the pandemic misdiagnosed hospitalisations as well as laboratory-monitoring drastically increased. This would introduce considerable variability in the time series analysis. At the same time, we believe that our findings are informative for the management of current and future flu seasons as well as that of COVID-19, given that the burden of COVID-19 on hospitals concurs with that of all (pre)existing diseases, including influenza. Providing a realistic estimate of the burden of influenza in terms of hospitalisations in ‘normal times’ may help to plan healthcare needs during and beyond the COVID-19 pandemic. Overall, the present study supports the need for increased testing for influenza in Italy to tackle the current underestimation of influenza burden. According to this study, we conclude that there is still a substantial burden of influenza on the elderly population and younger population in Italy, which needs to be addressed.

As a result, these findings on the clinical and economic burden of influenza should also provide further insights for policy evaluation and decision-making.

### Supplementary Information

**Additional file 1: Table A1.** Diagnosis of hospitalizations and related ICD-9-CM codes. **Table A2.** Hospital Discharge Record Variables. **Table A3.** Coefficient estimates of the negative binomial regression. Y=hospital admissions for all ICD9 codes considered; all ages; Italy. **Table A4.** Model specifications fit. Based on Y=hospital admissions for all ICD9 codes considered; all ages; season 2008/09-2018/19, Italy. **Table A5.** Seasonal Numbers of Hospitalizations, by ICD9-CM group. **Table A6.** Seasonal Numbers of Hospitalizations, by ICD9-CM group and age group. **Table A7.** Estimated numbers and rates per 100,000 people of excess hospitalization associated with influenza, for respiratory and cardiorespiratory group of diagnoses. Seasons 2008/09-2018/19, Italy. **Table A8.** Estimated Numbers and Rates per 100,000 people of excess hospitalization associated with influenza including code 487. Seasons 2008/09-2018/19, Italy. **Table A9.** Estimated numbers and rates per 100,000 people of excess hospitalization associated with influenza, by age and season for respiratory and cardiorespiratory diagnoses. **Table A10.** Observed Seasonal Numbers of Influenza-Hospitalizations and Hospitalisations associated with influenza (all ICD9 considered), by region. All ages. **Table A11.** Estimated Numbers and Rates per 100,000 People of Excess Hospitalization Associated with Influenza by Region, All ICD9 Diagnoses and Ages Considered. **Table A12.** Seasonal mean of incidence of ILI per 1000 patients among population aged 65 years and over, by region. **Table A13.** Rate per 100,000 people of hospitalizations with primary code 487 among people aged 65+, by region. **Figure A1.** Average rates per 100,000 people of excess hospitalization associated with influenza from seasons 2008/09 to 2018/19, by region. All ages. **Table A14.** Seasonal costs of influenza and influenza-associated hospitalisations. Season 2008/09-2018/19, Italy. **Table A15.** In hospital mortality from seasons 2008/09 to 2018/19, by different groups of diagnoses at hospital admission. All ages. **Table A16.** In hospital mortality from seasons 2008/09 to 2018/19, by region. All ages. **Table A17.** Estimates of numbers and rates per 100,000 people of excess in-hospital mortality associated with influenza, by region and season. All ages.

## Data Availability

The data that support the findings of this study are available from of the Italian Health Ministry but restrictions apply to the availability of these data, which were used under license for the current study, and so are not publicly available. Data may be obtained by a third party only by request to the Italian Ministry of Health. Statistical code will be made available upon request (email to corresponding author Benedetta Pongiglione: benedetta.pongiglione@unibocconi.it).

## Abbreviations

CIRI: Interuniversity Center for Influenza Research (Centro Interuniversitario per la Ricerca sull’Influenza)
HDR: Hospital Discharge Records
ICD-9-CM: International Classification of Diseases, Ninth Revision, Clinical Modification
ILI: Influenza-like illness
ISPRA: Istituto Superiore per la Protezione e Ricerca Ambientale
ISS: Italian National Institute of Health (Istituto Superiore di Sanità)
ISTAT: Institute for National Statistics
MMG: General Practitioner (Medici di Medicina Generale)
NHS: National Health Service
PLS: Primary-Care Paediatricians (Pediatri Libera Scelta)
SCIA: National System for the Collection, Processing, and Dissemination of Climate Data of Environmental Interest (Sistema nazionale per la raccolta, l’elaborazione e la diffusione di dati Climatici di Interesse Ambientale)

## Footnotes

1 With ‘observed hospitalizations’ it is meant hospitalizations that are actually recorded in the HDR with influenza ICD9 code, while ‘estimated hospitalizations’ are derived from the model described above, and as such estimated.
