# Supplementary material for: Excess hospitalizations and in-hospital mortality associated with seasonal influenza in Italy: a 11-year retrospective study
Source: BMC Infect Dis. 2024 Feb 20;24:227. doi: 10.1186/s12879-024-09071-z (PMC10877853; doi:10.1186/s12879-024-09071-z)
Supplement: Supplementary file 1 — Additional file 1: Table A1. Diagnosis of hospitalizations and related ICD-9-CM codes. Table A2. Hospital Discharge Record Variables. Table A3. Coefficient estimates of the negative binomial regression. Y=hospital admissions for all ICD9 codes considered; all ages; Italy. Table A4. Model specifications fit. Based on Y=hospital admissions for all ICD9 codes considered; all ages; season 2008/09-2018/19, Italy. Table A5. Seasonal Numbers of Hospitalizations, by ICD9-CM group. Table A6. Seasonal Numbers of Hospitalizations, by ICD9-CM group and age group. Table A7. Estimated numbers and rates per 100,000 people of excess hospitalization associated with influenza, for respiratory and cardiorespiratory group of diagnoses. Seasons 2008/09-2018/19, Italy. Table A8. Estimated Numbers and Rates per 100,000 people of excess hospitalization associated with influenza including code 487. Seasons 2008/09-2018/19, Italy. Table A9. Estimated numbers and rates per 100,000 people of excess hospitalization associated with influenza, by age and season for respiratory and cardiorespiratory diagnoses. Table A10. Observed Seasonal Numbers of Influenza-Hospitalizations and Hospitalisations associated with influenza (all ICD9 considered), by region. All ages. Table A11. Estimated Numbers and Rates per 100,000 People of Excess Hospitalization Associated with Influenza by Region, All ICD9 Diagnoses and Ages Considered. Table A12. Seasonal mean of incidence of ILI per 1000 patients among population aged 65 years and over, by region. Table A13. Rate per 100,000 people of hospitalizations with primary code 487 among people aged 65+, by region. Figure A1. Average rates per 100,000 people of excess hospitalization associated with influenza from seasons 2008/09 to 2018/19, by region. All ages. Table A14. Seasonal costs of influenza and influenza-associated hospitalisations. Season 2008/09-2018/19, Italy. Table A15. In hospital mortality from seasons 2008/09 to 2018/19, by different groups of diagnos [file 12879_2024_9071_MOESM1_ESM.docx]

Additional file of “*Excess hospitalizations and in-hospital mortality associated with seasonal influenza in Italy: a 11-year retrospective study*”

**Table A1.** Diagnosis of hospitalizations and related ICD-9-CM codes.

| **Code ICD-9-CM** | **Diagnosis** | **Classification** |
| --- | --- | --- |
| 040 | Other bacterial diseases | Other |
| 250 | Diabetes mellitus | Other |
| 322 | Meningitis of unspecified cause | Other |
| 323 | Encephalitis myelitis and encephalomyelitis | Other |
| 341 | Other demyelinating diseases of central nervous system | Other |
| 357 | Inflammatory and toxic neuropathy | Other |
| 382 | Suppurative and unspecified otitis media | Other |
| 410 | Acute myocardial infraction | Diseases of the circulatory system |
| 411 | Other acute ischemic heart diseases | Diseases of the circulatory system |
| 412 | Old myocardial infraction | Diseases of the circulatory system |
| 413 | Angina pectoris | Diseases of the circulatory system |
| 414 | Chronic ischemic heart disease, unspecified | Diseases of the circulatory system |
| 422 | Acute myocarditis | Diseases of the circulatory system |
| 427 | Cardiac arrhythmias | Diseases of the circulatory system |
| 428 | Heart failure | Diseases of the circulatory system |
| 430 | Nontraumatic subarachnoid hemorrhage | Diseases of the circulatory system |
| 431 | Nontraumatic intracerebral hemorrhage | Diseases of the circulatory system |
| 432 | Other and unspecified nontraumatic intracranial hemorrhage | Diseases of the circulatory system |
| 433 | Occlusion and stenosis of precerebral artery | Diseases of the circulatory system |
| 434 | Cerebral infarction due to thrombosis of carotid artery | Diseases of the circulatory system |
| 435 | Transient cerebral ischemic attacks and related syndromes | Diseases of the circulatory system |
| 437 | Cerebrovascular disease, unspecified | Diseases of the circulatory system |
| 438 | Vascular syndromes of brain in cerebrovascular diseases | Diseases of the circulatory system |
| 440 | Atherosclerosis | Diseases of the circulatory system |
| 460 | Acute nasopharyngitis | Diseases of the respiratory system |
| 461 | Acute sinusitis | Diseases of the respiratory system |
| 462 | Acute pharyngitis | Diseases of the respiratory system |
| 463 | Acute tonsillitis | Diseases of the respiratory system |
| 464 | Acute laryngitis | Diseases of the respiratory system |
| 465 | Acute upper respiratory infections of multiple or unspecified sites | Diseases of the respiratory system |
| 466 | Acute bronchitis and bronchiolitis | Diseases of the respiratory system |
| 480 | Viral pneumonia | Pneumonia and influenza |
| 481 | Pneumococcal pneumonia | Pneumonia and influenza |
| 482 | Other bacterial pneumonia | Pneumonia and influenza |
| 483 | Pneumonia due to other specified organism | Pneumonia and influenza |
| 484 | Pneumonia in infectious diseases classified elsewhere | Pneumonia and influenza |
| 485 | Bronchopneumonia, organism unspecified | Pneumonia and influenza |
| 486 | Pneumonia, organism unspecified | Pneumonia and influenza |
| 487 | Influenza | Influenza |
| 490 | Bronchitis, not specified as acute or chronic | Diseases of the respiratory system |
| 491 | Chronic bronchitis | Diseases of the respiratory system |
| 492 | Emphysema | Diseases of the respiratory system |
| 493 | Asthma | Diseases of the respiratory system |
| 494 | Bronchiectasis | Diseases of the respiratory system |
| 495 | Extrinsic allergic alveolitis | Diseases of the respiratory system |
| 496 | Chronic airway obstruction, not elsewhere classified | Diseases of the respiratory system |
| 500 | Coal workers' pneumoconiosis | Diseases of the respiratory system |
| 501 | Asbestosis | Diseases of the respiratory system |
| 502 | Pneumoconiosis due to other silica or silicates | Diseases of the respiratory system |
| 503 | Pneumoconiosis due to other inorganic dust | Diseases of the respiratory system |
| 504 | Pneumonopathy due to inhalation of other dust | Diseases of the respiratory system |
| 505 | Pneumoconiosis, unspecified | Diseases of the respiratory system |
| 506 | Respiratory conditions due to chemical fumes and vapors | Diseases of the respiratory system |
| 507 | Pneumonitis due to solids and liquids | Diseases of the respiratory system |
| 508 | Respiratory conditions due to other and unspecified external agents | Diseases of the respiratory system |
| 510 | Empyema | Diseases of the respiratory system |
| 511 | Pleurisy | Diseases of the respiratory system |
| 512 | Pneumothorax and air leak | Diseases of the respiratory system |
| 513 | Abscess of lung and mediastinum | Diseases of the respiratory system |
| 514 | Pulmonary congestion and hypostasis | Diseases of the respiratory system |
| 515 | Postinflammatory pulmonary fibrosis | Diseases of the respiratory system |
| 516 | Pulmonary alveolar proteinosis | Diseases of the respiratory system |
| 518 | Other diseases of pulmonary system | Diseases of the respiratory system |
| 728 | Disorders of muscle ligament and fascia | Other |
| 729 | Other disorders of soft tissues | Other |
| 747 | Other congenital anomalies of circulatory system | Other |

ICD-9-CM =International Classification of Diseases, Ninth Revision

**Table A2.** Hospital Discharge Record Variables

| **Variable** | **Values** |
| --- | --- |
| Code of region of admission | "010" = Piemonte; "020" = Val d'Aosta; "030" = Lombardia; "041" = P.A. di Bolzano; "042" = P.A. di Trento; "050" = Veneto; "060" = Friuli Venezia Giulia; "070" = Liguria; "080" = Emilia Romagna; "090" = Toscana; "100" = Umbria; "110" = Marche; "120" = Lazio; "130" = Abruzzo; "140" = Molise; "150" = Campania; "160" = Puglia; "170" = Basilicata; "180" = Calabria; "190" = Sicilia; "200" = Sardegna |
| Age in years | Integer numbers from 0 to 124 |
| Age class | "0" = age not calculable; "1" = 0 years; "2" = 1-4 years; "3" = 5-13 years; "4" = 14 years; "5" = 15-24 years; "6" = 25-44 years; "7" = 45-64 years; "8" = 65-74 years; "9" = 75 years and over |
| Admission regime (to identify ordinary admissions) | 1 = Ordinary hospitalisation  2 = Day hospitalisation |
| Date of admission | format: yyyyymmdd |
| Date of discharge | format: yyyyymmdd |
| Days of hospitalization | Integer continuous number. Indicates the length of stay in ordinary care if admission regime = "1"; indicates the number of Day Hospital admissions if admission regime = "2" |
| Mode of discharge (to identify in-hospital mortality) | 01 = death;  02 = ordinary discharge to the patient's home  03 = ordinary discharge to a territorial residential facility  04 = discharge to the patient's home with activation of home assistance  05 = voluntary discharge  06 = transfer to another acute care institution, public or private  07 = transfer to another in-patient regime (day or ordinary hospitalisation) or to another type of in-patient activity (acute, rehabilitation, long-term care) within the same healthcare institution;  08 = Transfer to a public or private rehabilitation institution.  09 = Ordinary discharge with activation of ADI |
| Main diagnosis | ICD-9-CM code 2007 version |
| Secondary diagnosis 1 | ICD-9-CM code 2007 version |
| Secondary diagnosis 2 | ICD-9-CM code 2007 version |
| Secondary diagnosis 3 | ICD-9-CM code 2007 version |
| Secondary diagnosis 4 | ICD-9-CM code 2007 version |
| Secondary diagnosis 5 | ICD-9-CM code 2007 version |
| Diagnosis Related Groups (DRG) code | DRG code version 24 |
| Major Diagnostic Category (MDC) code | code MDC version 24 |

ICD-9-CM =International Classification of Diseases, Ninth Revision

**Table A3.** Coefficient estimates of the negative binomial regression. Y=hospital admissions for all ICD9 codes considered; all ages; Italy.

|  | **β** | **SE** | **95% CI** | **p-value** |
| --- | --- | --- | --- | --- |
| ILI incidence | 0.008 | 0.00124 | (0.005 - 0.010) | <0.001 |
| Temperature | -0.004 | 0.00135 | (-0.006 - -0.0009) | 0.009 |
| N week | -0.0002 | 1.61E-05 | (-0.0003 - -0.0002) | <0.001 |
| Cosine | -0.052 | 0.0122 | (-0.076 - -0.028) | <0.001 |
| Sine | -0.079 | 0.00705 | (-0.093 - -0.066) | <0.001 |
| Constant | 10.29 | 0.0184 | (10.25 - 10.32) | <0.001 |

ILI=influenza-like illness; SE=standard error; CI=confidence interval

**Table A4.** Model specifications fit. Based on Y=hospital admissions for all ICD9 codes considered; all ages; season 2008/09-2018/19, Italy.

| N model | Model content | N | ll(null) | ll(model) | df | AIC | BIC |
| --- | --- | --- | --- | --- | --- | --- | --- |
| **M1** | **0 lag** | **574** | **-5468.77** | **-5061.85** | **7** | **10137.71** | **10168.18** |
| M2 | 1 lag | 574 | -5468.77 | -5064.73 | 7 | 10143.45 | 10173.92 |
| M3 | 2 lag | 574 | -5468.77 | -5070.02 | 7 | 10154.05 | 10184.52 |
| M4 | 3 lag | 574 | -5468.77 | -5075.79 | 7 | 10165.59 | 10196.06 |
| M5 | 3w ma centred | 574 | -5468.77 | -5062.86 | 7 | 10139.72 | 10170.19 |
| M6 | 3w ma lagged | 574 | -5468.77 | -5064.51 | 7 | 10143.02 | 10173.49 |

M= model; ll=log-likelihood; df=degrees of freedom; AIC= Akaike information criterion; BIC= Bayesian information criterion; ma=moving average

**Table A5.** Seasonal Numbers of Hospitalizations, by ICD9-CM group

| Season* | Influenza  (ICD9 diagnosis 487) | Pneumonia and Influenza  (ICD9 diagnosis 480-487) | | Diseases of the respiratory system  (ICD9 diagnosis 480-487; 460–466, 480–487, 490–496, 500–508, 510–516, 518) | | Cardiorespiratory diseases  (ICD9 diagnosis 480-487; 460–466, 480–487, 490–496, 500–508, 510–516, 518; 410–414, 422, 427, 428, 430–435, 437, 438, 440) | |  |
| --- | --- | --- | --- | --- | --- | --- | --- | --- |
|  | any listed | | primary | | primary | | primary | |
| 2008/2009 | 5,920 | | 127,312 | | 483,258 | | 1,396,622 | |
| 2009/2010 | 14,525 | | 144,514 | | 496,018 | | 1,410,866 | |
| 2010/2011 | 7,002 | | 141,066 | | 487,077 | | 1,372,979 | |
| 2011/2012 | 4,876 | | 130,299 | | 470,206 | | 1,338,078 | |
| 2012/2013 | 6,097 | | 131,968 | | 466,217 | | 1,305,492 | |
| 2013/2014 | 4,232 | | 137,924 | | 467,984 | | 1,295,158 | |
| 2014/2015 | 7,568 | | 147,662 | | 490,921 | | 1,311,733 | |
| 2015/2016 | 4,690 | | 136,848 | | 471,490 | | 1,287,533 | |
| 2016/2017 | 6,836 | | 145,294 | | 489,022 | | 1,274,892 | |
| 2017/2018 | 10,224 | | 147,381 | | 492,004 | | 1,259,442 | |
| 2018/2019 | 11,316 | | 156,833 | | 507,980 | | 1,261,662 | |
| *Total* | *83,286* | | *1,547,101* | | *5,322,177* | | *14,514,457* | |
| *11-season mean* | *7571.5* | | *140645.5* | | *483834.3* | | *1319496.1* | |
| *3-season mean*§ | *9,459* | | *149,836* | | *496,335* | | *1,265,332* | |

*Each season extends from week 27 of one year to week 26 of the following year

§ Includes the most recent seasons, namely 2016/17, 2017/18, and 2018/19

**Table A6.** Seasonal Numbers of Hospitalizations, by ICD9-CM group and age group

| Season* | Respiratory diseases | | | | Cardiorespiratory diseases | | | |
| --- | --- | --- | --- | --- | --- | --- | --- | --- |
|  | 0-4 years | 5-14 years | 15-64 years | 65+ years | 0-4 years | 5-14 years | 15-64 years | 65+ years |
| 2008/2009 | 67,883 | 16,121 | 93,597 | 302,142 | 69,026 | 17,050 | 305,200 | 1,005,106 |
| 2009/2010 | 66,297 | 19,031 | 99,878 | 301,588 | 67,395 | 20,001 | 313,222 | 1,009,775 |
| 2010/2011 | 62,855 | 17,520 | 97,878 | 304,798 | 63,909 | 18,590 | 303,979 | 986,490 |
| 2011/2012 | 55,045 | 12,513 | 86,144 | 313,870 | 56,075 | 13,631 | 285,312 | 983,052 |
| 2012/2013 | 53,371 | 12,475 | 88,522 | 308,400 | 54,440 | 13,713 | 280,027 | 957,312 |
| 2013/2014 | 50,769 | 15,254 | 87,273 | 312,240 | 51,757 | 16,498 | 272,601 | 954,301 |
| 2014/2015 | 51,544 | 13,533 | 89,746 | 332,288 | 52,582 | 14,843 | 274,054 | 970,250 |
| 2015/2016 | 48,883 | 11,637 | 83,742 | 324,746 | 49,873 | 12,960 | 266,998 | 957,702 |
| 2016/2017 | 48,569 | 10,954 | 83,288 | 343,083 | 49,601 | 12,285 | 260,152 | 952,854 |
| 2017/2018 | 46,317 | 9,942 | 86,377 | 344,772 | 47,257 | 11,281 | 261,879 | 939,025 |
| 2018/2019 | 46,792 | 12,454 | 89,793 | 354,283 | 47,681 | 13,833 | 262,650 | 937,481 |
| *Total* | *598,325* | *151,434* | *986,238* | *3,542,210* | *609,596* | *164,685* | *3,086,074* | *10,653,348* |
| *11-season mean* | *54,393* | *13,767* | *89,658* | *322,019* | *55,418* | *14,971* | *280,552* | *968,486* |
| *3-season mean*§ | *47,226* | *11,117* | *86,486* | *347,379* | *48,180* | *12,466* | *261,560* | *943,120* |

*Each season extends from week 27 of one year to week 26 of the following year

§Includes the most recent seasons, namely 2016/17, 2017/18, and 2018/19

**Table A7**. Estimated numbers and rates per 100,000 people of excess hospitalization associated with influenza, for respiratory and cardiorespiratory group of diagnoses. Seasons 2008/09-2018/19, Italy

| Season* | Respiratory diagnoses | | Cardiorespiratory diagnoses | |
| --- | --- | --- | --- | --- |
|  | N | Rate^§^ | N | Rate^§^ |
| 2008/09 | 15,936.4 | 26.9 | 15,684.2 | 26.5 |
| 2009/10 | 19,430.5 | 32.6 | 19,576.8 | 32.9 |
| 2010/11 | 22,940.7 | 38.3 | 21,725.5 | 36.3 |
| 2011/12 | 19,130.2 | 31.9 | 18,097.3 | 30.1 |
| 2012/13 | 24,033.5 | 39.9 | 22,207.6 | 36.9 |
| 2013/14 | 17,466.5 | 29.0 | 16,120.4 | 26.7 |
| 2014/15 | 24,380.3 | 40.4 | 21,927.9 | 36.4 |
| 2015/16 | 18,258.8 | 30.3 | 16,499.0 | 27.4 |
| 2016/17 | 20,538.1 | 34.2 | 18,322.1 | 30.5 |
| 2017/18 | 34,437.6 | 57.4 | 29,398.1 | 49.0 |
| 2018/19 | 32,038.8 | 53.5 | 27,176.8 | 45.4 |
| Total | 248591.4 |  | 226735.7 |  |
| 11-season mean | 22599.2 | 37.7 | 20612.3 | 34.4 |
| 3-season mean^¥^ | 29004.8 | 48.4 | 24965.7 | 41.6 |

*Each season extends from week 27 of one year to week 26 of the following year

§ Every 100,000 inhabitants

^¥^Includes the most recent seasons, namely 2016/17, 2017/18, and 2018/19

**Table A8.** Estimated Numbers and Rates per 100,000 people of excess hospitalization associated with influenza including code 487. Seasons 2008/09-2018/19, Italy

| Season* | Respiratory diagnoses (including influenza 487) | | All ICD9 considered (including 487) | |
| --- | --- | --- | --- | --- |
|  | N | Rate^§^ | N | Rate^§^ |
| 2008/09 | 18,542.70 | 31.3 | 19,561.50 | 33.0 |
| 2009/10 | 22,710.50 | 38.1 | 24,463.40 | 41.1 |
| 2010/11 | 26,716.10 | 44.7 | 27,021.10 | 45.2 |
| 2011/12 | 22,253.50 | 37.1 | 22,484.30 | 37.5 |
| 2012/13 | 27,992.80 | 46.5 | 27,575.50 | 45.8 |
| 2013/14 | 20,314.10 | 33.7 | 20,015.40 | 33.2 |
| 2014/15 | 28,571.80 | 47.4 | 27,374.60 | 45.4 |
| 2015/16 | 20,773.60 | 34.5 | 20,006.10 | 33.2 |
| 2016/17 | 23,994.20 | 39.9 | 22,737.50 | 37.8 |
| 2017/18 | 40,165.40 | 66.9 | 36,277.30 | 60.5 |
| 2018/19 | 37,274.40 | 62.3 | 33,463.40 | 55.9 |
| *Total* | 289309.1 |  | 280980.1 |  |
| 11-season mean | 26300.8 | 43.8 | 25543.6 | 42.6 |
| 3-season mean^¥^ | 33811.3 | 56.4 | 30826.1 | 51.4 |

*Each season extends from week 27 of one year to week 26 of the following year

^§^ Every 100,000 inhabitants

^¥^Includes the most recent seasons, namely 2016/17, 2017/18, and 2018/19

**Table A9.** Estimated numbers and rates per 100,000 people of excess hospitalization associated with influenza, by age and season for respiratory and cardiorespiratory diagnoses

| Season* | Respiratory diseases | | | | | | | | Cardiorespiratory diseases | | | | | | | |
| --- | --- | --- | --- | --- | --- | --- | --- | --- | --- | --- | --- | --- | --- | --- | --- | --- |
|  | 0-4 years | | 5-14 years | | 15-64 years | | 65+ years | | 0-4 years | | 5-14 years | | 15-64 years | | 65+ years | |
|  | N | Rate^¥^ | N | Rate^¥^ | N | Rate^¥^ | N | Rate^¥^ | N | Rate^¥^ | N | Rate^¥^ | N | Rate^¥^ | N | Rate^¥^ |
| 2008/09 | 2010.6 | 71.6 | 513.4 | 9.3 | 3407.3 | 8.8 | 13852.7 | 115.8 | 2062.1 | 73.4 | 511.0 | 9.2 | 3131.5 | 8.0 | 13489.4 | 112.8 |
| 2009/10 | 1721.5 | 61.0 | 880.2 | 15.8 | 3464.7 | 8.9 | 9734.0 | 80.5 | 1779.4 | 63.1 | 884.5 | 15.9 | 3168.5 | 8.1 | 9697.3 | 80.2 |
| 2010/11 | 2493.7 | 88.5 | 705.6 | 12.6 | 4722.6 | 12.1 | 11118.5 | 91.1 | 2563.3 | 90.9 | 710.8 | 12.7 | 4243.5 | 10.8 | 10538.3 | 86.4 |
| 2011/12 | 2102.0 | 75.1 | 437.0 | 7.8 | 4167.5 | 10.6 | 15848.8 | 127.9 | 2160.4 | 77.2 | 443.1 | 7.9 | 3751.1 | 9.6 | 14463.4 | 116.8 |
| 2012/13 | 2295.9 | 82.8 | 627.7 | 11.1 | 5103.4 | 13.0 | 16007.7 | 126.7 | 2362.7 | 85.2 | 640.1 | 11.3 | 4524.8 | 11.6 | 14334.6 | 113.5 |
| 2013/14 | 1687.2 | 61.7 | 346.6 | 6.1 | 3812.2 | 9.8 | 13711.7 | 106.6 | 1740.0 | 63.6 | 357.6 | 6.3 | 3416.4 | 8.8 | 12045.3 | 93.7 |
| 2014/15 | 1982.2 | 74.1 | 498.3 | 8.8 | 5470.1 | 14.1 | 20756.4 | 158.7 | 2044.5 | 76.5 | 516.2 | 9.1 | 4772.7 | 12.3 | 17546.7 | 134.1 |
| 2015/16 | 1606.4 | 61.7 | 448.8 | 7.9 | 3608.1 | 9.3 | 11882.5 | 89.7 | 1659.7 | 63.8 | 468.1 | 8.3 | 3226.3 | 8.3 | 10154.9 | 76.6 |
| 2016/17 | 1578.2 | 62.4 | 324.6 | 5.8 | 4570.5 | 11.9 | 23402.2 | 174.8 | 1631.1 | 64.5 | 341.7 | 6.1 | 3978.1 | 10.3 | 18776.3 | 140.2 |
| 2017/18 | 2682.4 | 109.4 | 524.5 | 9.4 | 7335.0 | 19.1 | 37073.4 | 274.3 | 2774.8 | 113.1 | 554.8 | 9.9 | 6136.1 | 16.0 | 28205.7 | 208.7 |
| 2018/19 | 2482.5 | 104.5 | 478.9 | 8.6 | 6823.1 | 17.8 | 29663.6 | 217.6 | 2569.3 | 108.1 | 509.4 | 9.2 | 5752.4 | 15.0 | 22947.9 | 168.4 |
| Total | 22642.7 |  | 5785.6 |  | 52484.4 |  | 203052.0 |  | 23347.3 |  | 5937.4 |  | 46101.4 |  | 172200.0 |  |
| 11-season mean | 2058.4 | 77.5 | 526.0 | 9.4 | 4771.3 | 12.3 | 18459.2 | 142.2 | 2122.5 | 80.0 | 539.8 | 9.6 | 4191.0 | 10.8 | 15654.5 | 121.0 |
| 3-season mean^§^ | 2247.7 | 92.1 | 442.7 | 7.9 | 6242.9 | 16.3 | 30046.4 | 222.2 | 2325.1 | 95.3 | 468.7 | 8.4 | 5288.9 | 13.8 | 23310.0 | 172.4 |

* Each season extends from week 27 of one year to week 26 of the following year

¥ Every 100,000 inhabitants

^§^ Includes the most recent seasons, namely 2016/17, 2017/18, and 2018/19

**Table A10.** Observed Seasonal Numbers of Influenza-Hospitalizations and Hospitalisations associated with influenza (all ICD9 considered), by region. All ages

| Season* | Influenza (ICD9 diagnosis 487) | | | | | | | | | | | | | | | | | | | | |
| --- | --- | --- | --- | --- | --- | --- | --- | --- | --- | --- | --- | --- | --- | --- | --- | --- | --- | --- | --- | --- | --- |
|  | PM | VA | LO | BZ | TR | VE | FVG | LG | ER | TS | UM | MA | LZ | AB | MO | CA | PU | BA | CB | SI | SA |
| 2008/09 | 149 | 1 | 270 | 92 | 23 | 160 | 40 | 35 | 221 | 119 | 62 | 69 | 332 | 103 | 7 | 393 | 359 | 64 | 194 | 465 | 246 |
| 2009/10 | 453 | 7 | 928 | 162 | 50 | 432 | 75 | 225 | 570 | 459 | 162 | 274 | 956 | 275 | 36 | 1086 | 1028 | 82 | 378 | 1069 | 347 |
| 2010/11 | 228 | 8 | 410 | 121 | 21 | 245 | 39 | 63 | 295 | 181 | 81 | 137 | 274 | 57 | 16 | 367 | 583 | 44 | 220 | 446 | 186 |
| 2011/12 | 176 | 4 | 270 | 51 | 19 | 130 | 27 | 41 | 175 | 125 | 47 | 39 | 196 | 53 | 5 | 224 | 272 | 18 | 151 | 433 | 175 |
| 2012/13 | 119 | 6 | 356 | 85 | 26 | 158 | 70 | 38 | 319 | 121 | 50 | 59 | 266 | 66 | 10 | 336 | 544 | 37 | 203 | 477 | 103 |
| 2013/14 | 79 | 3 | 224 | 36 | 18 | 67 | 56 | 28 | 175 | 51 | 40 | 35 | 246 | 32 | 6 | 255 | 358 | 14 | 267 | 324 | 133 |
| 2014/15 | 121 | 10 | 512 | 72 | 48 | 344 | 32 | 69 | 373 | 133 | 73 | 77 | 313 | 35 | 10 | 314 | 369 | 23 | 292 | 464 | 123 |
| 2015/16 | 91 | 3 | 334 | 44 | 37 | 208 | 18 | 37 | 256 | 61 | 43 | 36 | 231 | 37 | 7 | 122 | 250 | 13 | 173 | 383 | 98 |
| 2016/17 | 85 | 5 | 524 | 76 | 26 | 329 | 36 | 65 | 474 | 114 | 56 | 29 | 299 | 34 | 2 | 144 | 232 | 14 | 182 | 271 | 131 |
| 2017/18 | 176 | 8 | 842 | 132 | 27 | 375 | 88 | 154 | 728 | 161 | 73 | 55 | 427 | 86 | 4 | 150 | 299 | 13 | 224 | 343 | 231 |
| 2018/19 | 293 | 3 | 841 | 177 | 62 | 450 | 181 | 116 | 771 | 214 | 63 | 47 | 316 | 50 | 3 | 153 | 300 | 10 | 190 | 269 | 133 |
| Total | 1970 | 58 | 5511 | 1048 | 357 | 2898 | 662 | 871 | 4357 | 1739 | 750 | 857 | 3856 | 828 | 106 | 3544 | 4594 | 332 | 2474 | 4944 | 1906 |
| 11-season mean | 179.1 | 5.3 | 501 | 95.3 | 32.5 | 263.5 | 60.2 | 79.2 | 396.1 | 158.1 | 68.2 | 77.9 | 350.5 | 75.3 | 9.6 | 322.2 | 417.6 | 30.2 | 224.9 | 449.5 | 173.3 |
| 3-season mean^§^ | 184.7 | 5.3 | 735.7 | 128.3 | 38.3 | 384.7 | 101.7 | 111.7 | 657.7 | 163.0 | 64.0 | 43.7 | 347.3 | 56.7 | 3.0 | 149.0 | 277.0 | 12.3 | 198.7 | 294.3 | 165.0 |

| Season* | All ICD9 considered | | | | | | | | | | | | | | | | | | | | |
| --- | --- | --- | --- | --- | --- | --- | --- | --- | --- | --- | --- | --- | --- | --- | --- | --- | --- | --- | --- | --- | --- |
|  | PM | VA | LO | BZ | TR | VE | FVG | LG | ER | TS | UM | MA | LZ | AB | MO | CA | PU | BA | CB | SI | SA |
| 2008/09 | 94103 | 2547 | 247771 | 13174 | 12738 | 109381 | 29965 | 42117 | 123146 | 94185 | 21743 | 41331 | 134733 | 35655 | 10450 | 135884 | 112618 | 13961 | 48225 | 125829 | 33452 |
| 2009/10 | 96980 | 2636 | 248580 | 13456 | 12084 | 109407 | 29365 | 42692 | 123985 | 96360 | 23081 | 41684 | 139552 | 34427 | 10410 | 135141 | 113531 | 14808 | 45782 | 126554 | 35257 |
| 2010/11 | 95372 | 2716 | 242638 | 13266 | 12030 | 106428 | 28753 | 41645 | 121933 | 94089 | 22514 | 40935 | 130632 | 33698 | 10387 | 131231 | 105425 | 14509 | 47830 | 121643 | 34239 |
| 2011/12 | 94874 | 2876 | 238654 | 13221 | 12610 | 103382 | 28526 | 40456 | 120045 | 93746 | 22009 | 38565 | 126237 | 33557 | 9466 | 125618 | 97699 | 13627 | 44560 | 117407 | 33871 |
| 2012/13 | 93684 | 2911 | 231607 | 12887 | 12691 | 102028 | 28175 | 40082 | 117785 | 88583 | 21657 | 37172 | 126634 | 33752 | 9567 | 126799 | 91874 | 13267 | 37527 | 114343 | 32171 |
| 2013/14 | 91708 | 3261 | 229121 | 12598 | 12470 | 101267 | 27681 | 39561 | 116013 | 88742 | 22250 | 35887 | 124650 | 33458 | 9577 | 125677 | 93396 | 13728 | 37374 | 111222 | 31820 |
| 2014/15 | 93330 | 3321 | 237147 | 12687 | 13092 | 103873 | 26553 | 39263 | 118699 | 91661 | 22636 | 35927 | 125509 | 33785 | 9929 | 124230 | 92769 | 13592 | 36648 | 110287 | 32283 |
| 2015/16 | 91949 | 3316 | 236669 | 12319 | 13013 | 104184 | 27896 | 39058 | 114054 | 88357 | 21694 | 34863 | 123498 | 33013 | 9230 | 121869 | 90243 | 12967 | 35445 | 106899 | 31706 |
| 2016/17 | 88035 | 3342 | 234035 | 12284 | 12578 | 102634 | 28560 | 39199 | 117962 | 88793 | 21571 | 34566 | 123809 | 32952 | 8563 | 118017 | 87889 | 13245 | 35047 | 103360 | 30751 |
| 2017/18 | 90238 | 3215 | 228784 | 12338 | 11957 | 101791 | 28049 | 38884 | 118165 | 87646 | 21787 | 33540 | 122281 | 32623 | 8500 | 115520 | 85705 | 13183 | 34320 | 100917 | 31595 |
| 2018/19 | 90190 | 3201 | 227738 | 11927 | 12300 | 101570 | 28395 | 39412 | 120234 | 88797 | 21830 | 34204 | 121029 | 31336 | 8432 | 115993 | 85095 | 13015 | 35349 | 99776 | 30807 |
| Total | 1020463 | 33342 | 2602744 | 140157 | 137563 | 1145945 | 311918 | 442369 | 1312021 | 1000959 | 242772 | 408674 | 1398564 | 368256 | 104511 | 1375979 | 1056244 | 149902 | 438107 | 1238237 | 357952 |
| 11-season ean | 92769.4 | 3031.1 | 236613.1 | 12741.5 | 12505.7 | 104176.8 | 28356.2 | 40215.4 | 119274.6 | 90996.3 | 22070.2 | 37152.2 | 127142.2 | 33477.8 | 9501.0 | 125089.0 | 96022.2 | 13627.5 | 39827.9 | 112567 | 32541.1 |
| 3-season mean^§^ | 89487.7 | 3252.7 | 230185.7 | 12183.0 | 12278.3 | 101998.3 | 28334.7 | 39165.0 | 118787.0 | 88412.0 | 21729.3 | 34103.3 | 122373.0 | 32303.7 | 8498.3 | 116510.0 | 86229.7 | 13147.7 | 34905.3 | 101351.0 | 31051.0 |

PM=Piedomont; VA=Valle d’Aosta; LO=Lombardy; BZ=Bolzano; TR=trento; VE=Veneto; FVG=Friuli Venezia Giulia; LG=Liguria; ER=Emilia Romagna; TS=Tuscany; UM=Umbria; MA=Marche; LZ=Lazio; AB=Abruzzo; MO=Molise; CA=Campania; PU=Puglia; BA=Basilicata; CB=Calabria; SI=Sicily; SA=Sardinia

*Each season extends from week 27 of one year to week 26 of the following year

^§^ Includes the most recent seasons, namely 2016/17, 2017/18, and 2018/19

**Table A11.** Estimated Numbers and Rates per 100,000 People of Excess Hospitalization Associated with Influenza by Region, All ICD9 Diagnoses and Ages Considered

| Season* | PM | | VA | | LO | | BZ | | TR | | VE | | FVG | | LG | | ER | | TS | |
| --- | --- | --- | --- | --- | --- | --- | --- | --- | --- | --- | --- | --- | --- | --- | --- | --- | --- | --- | --- | --- |
|  | N | Rate^§^ | N | Rate^§^ | N | Rate^§^ | N | Rate^§^ | N | Rate^§^ | N | Rate^§^ | N | Rate^§^ | N | Rate^§^ | N | Rate^§^ | N | Rate^§^ |
| 2008/2009 | 1669.8 | 38.2 | 92.2 | 73.1 | 1761.9 | 18.4 | 490.4 | 99.3 | 234.5 | 45.7 | 1669.1 | 34.6 | 253.8 | 20.8 | 774.9 | 48.8 | 1129.4 | 26.4 | 1321.3 | 36.0 |
| 2009/2010 | 2025.3 | 46.1 | 22.1 | 17.5 | 2533.5 | 26.3 | 475.4 | 95.4 | 234.8 | 45.2 | 1634.6 | 33.7 | 335.1 | 27.3 | 755.3 | 47.5 | 1673.6 | 38.7 | 1445.9 | 39.1 |
| 2010/2011 | 2807.9 | 63.7 | 153.0 | 120.4 | 2116.0 | 21.8 | 411.4 | 81.9 | 303.6 | 58.0 | 2359.3 | 48.4 | 356.4 | 29.1 | 864.8 | 54.3 | 1724.4 | 39.6 | 1563.1 | 42.1 |
| 2011/2012 | 2671.9 | 60.5 | 65.6 | 51.5 | 2283.0 | 23.3 | 326.8 | 64.6 | 367.7 | 69.7 | 1963.4 | 40.2 | 232.0 | 18.9 | 724.8 | 45.5 | 1427.6 | 32.6 | 1627.6 | 43.6 |
| 2012/2013 | 3036.2 | 68.7 | 64.1 | 50.2 | 2368.9 | 24.1 | 333.7 | 65.5 | 366.0 | 68.9 | 2102.5 | 43.0 | 310.6 | 25.4 | 908.9 | 57.2 | 2079.8 | 47.2 | 1777.2 | 47.5 |
| 2013/2014 | 1858.0 | 42.0 | 43.2 | 33.8 | 1640.5 | 16.6 | 231.3 | 45.0 | 234.2 | 43.8 | 1323.5 | 27.0 | 168.5 | 13.8 | 597.7 | 37.7 | 1377.7 | 31.1 | 1152.4 | 30.8 |
| 2014/2015 | 3065.1 | 69.5 | 88.1 | 68.7 | 2281.7 | 22.9 | 378.5 | 73.3 | 453.2 | 84.3 | 2160.0 | 44.0 | 258.5 | 21.1 | 797.3 | 50.6 | 2147.0 | 48.4 | 1695.4 | 45.3 |
| 2015/2016 | 2321.4 | 52.8 | 29.0 | 22.7 | 1946.7 | 19.6 | 197.4 | 38.0 | 425.0 | 78.9 | 1751.7 | 35.8 | 205.3 | 16.8 | 600.2 | 38.4 | 1774.7 | 40.0 | 990.2 | 26.5 |
| 2016/2017 | 2855.7 | 65.2 | 67.7 | 53.3 | 2048.1 | 20.6 | 305.0 | 58.5 | 482.2 | 89.3 | 1878.5 | 38.4 | 297.2 | 24.5 | 774.8 | 49.8 | 1912.1 | 43.1 | 1543.3 | 41.4 |
| 2017/2018 | 3162.7 | 72.5 | 53.6 | 42.4 | 4372.3 | 43.8 | 391.5 | 74.6 | 528.3 | 97.6 | 2313.9 | 47.4 | 439.0 | 36.2 | 1543.6 | 99.8 | 2238.1 | 50.4 | 2542.6 | 68.4 |
| 2018/2019 | 2944.4 | 67.9 | 38.2 | 30.4 | 4087.2 | 40.9 | 301.5 | 57.0 | 470.4 | 86.7 | 2160.6 | 44.2 | 385.3 | 31.8 | 1210.3 | 78.7 | 1999.4 | 44.9 | 2859.6 | 77.1 |
| Total | 28418.4 |  | 716.7 |  | 27439.8 |  | 3843.1 |  | 4099.9 |  | 21317.0 |  | 3241.7 |  | 9552.4 |  | 19483.8 |  | 18518.7 |  |
| 11-season mean | 2583.5 | 58.8 | 65.2 | 51.3 | 2494.5 | 25.3 | 349.4 | 68.5 | 372.7 | 69.8 | 1937.9 | 39.7 | 294.7 | 24.2 | 868.4 | 55.3 | 1771.3 | 40.2 | 1683.5 | 45.3 |
| 3-season mean^¥^ | 2987.6 | 68.5 | 53.2 | 42.0 | 3502.5 | 35.1 | 332.7 | 63.4 | 493.6 | 91.2 | 2117.6 | 43.4 | 373.8 | 30.8 | 1176.2 | 76.1 | 2049.9 | 46.1 | 2315.2 | 62.3 |

| Season* | UM | | MA | | LZ | | AB | | MO | | CA | | PU | | BA | | CB | | SI | | SA | |
| --- | --- | --- | --- | --- | --- | --- | --- | --- | --- | --- | --- | --- | --- | --- | --- | --- | --- | --- | --- | --- | --- | --- |
|  | N | Rate^§^ | N | Rate^§^ | N | Rate^§^ | N | Rate^§^ | N | Rate^§^ | N | Rate^§^ | N | Rate^§^ | N | Rate^§^ | N | Rate^§^ | N | Rate^§^ | N | Rate^§^ |
| 2008/09 | 104.5 | 11.9 | 446.2 | 29.1 | 1346.0 | 24.8 | 194.7 | 14.8 | 28.5 | 9.0 | 1131.5 | 19.5 | 987.4 | 24.2 | 40.2 | 6.9 | 50.3 | 2.5 | 1020.9 | 20.3 | 66.9 | 4.1 |
| 2009/10 | 99.8 | 11.3 | 545.3 | 35.3 | 1560.9 | 28.4 | 511.1 | 38.6 | 40.7 | 12.9 | 1410.6 | 24.3 | 1288.3 | 31.5 | 78.7 | 13.5 | 47.8 | 2.4 | 1744.6 | 34.6 | 88.8 | 5.4 |
| 2010/11 | 139.0 | 15.7 | 478.6 | 30.9 | 1874.1 | 33.8 | 429.2 | 32.3 | 53.9 | 17.1 | 1816.0 | 31.2 | 1209.7 | 29.5 | 46.0 | 7.9 | 70.0 | 3.5 | 1699.9 | 33.6 | 90.8 | 5.5 |
| 2011/12 | 109.1 | 12.3 | 364.6 | 23.5 | 1367.4 | 24.5 | 270.9 | 20.4 | 31.9 | 10.2 | 1412.4 | 24.2 | 1103.6 | 26.9 | 45.9 | 7.9 | 78.5 | 4.0 | 1884.4 | 37.2 | 123.3 | 7.4 |
| 2012/13 | 137.0 | 15.4 | 607.6 | 39.2 | 1786.1 | 31.7 | 337.6 | 25.3 | 36.6 | 11.7 | 1984.6 | 34.1 | 1179.8 | 28.8 | 104.5 | 18.1 | 95.4 | 4.9 | 1799.2 | 35.6 | 116.8 | 7.1 |
| 2013/14 | 97.8 | 11.0 | 452.6 | 29.2 | 1280.0 | 22.5 | 230.7 | 17.3 | 38.7 | 12.4 | 1348.3 | 23.2 | 876.0 | 21.4 | 65.9 | 11.4 | 89.5 | 4.6 | 1495.3 | 29.6 | 94.8 | 5.7 |
| 2014/15 | 146.5 | 16.5 | 614.2 | 39.7 | 1801.4 | 31.4 | 304.6 | 22.9 | 29.0 | 9.3 | 1859.8 | 32.0 | 1272.5 | 31.3 | 104.1 | 18.1 | 88.8 | 4.5 | 1539.5 | 30.6 | 126.0 | 7.6 |
| 2015/16 | 113.8 | 12.8 | 416.7 | 27.0 | 1420.3 | 24.7 | 236.8 | 17.9 | 15.3 | 4.9 | 1359.3 | 23.4 | 863.8 | 21.3 | 164.1 | 28.7 | 45.4 | 2.3 | 869.7 | 17.3 | 125.0 | 7.6 |
| 2016/17 | 133.7 | 15.1 | 352.9 | 23.0 | 1593.7 | 27.6 | 222.1 | 16.9 | 25.3 | 8.2 | 1404.7 | 24.3 | 952.1 | 23.6 | 100.7 | 17.7 | 12.5 | 0.6 | 1393.1 | 27.9 | 95.3 | 5.8 |
| 2017/18 | 230.6 | 26.2 | 526.9 | 34.5 | 1766.3 | 30.6 | 572.6 | 43.7 | 58.4 | 19.0 | 1521.0 | 26.4 | 2152.2 | 53.6 | 193.8 | 34.3 | 149.8 | 7.8 | 2762.9 | 55.7 | 121.3 | 7.4 |
| 2018/19 | 394.6 | 45.1 | 438.2 | 28.8 | 1777.9 | 30.8 | 547.0 | 42.0 | 24.5 | 8.0 | 1457.9 | 25.3 | 1861.2 | 46.7 | 34.1 | 6.1 | 255.2 | 13.3 | 2920.2 | 59.3 | 203.8 | 12.5 |
| Total | 1706.3 |  | 5243.9 |  | 17574.0 |  | 3857.4 |  | 382.9 |  | 16705.9 |  | 13746.6 |  | 977.9 |  | 983.1 |  | 19129.6 |  | 1253.0 |  |
| 11-season mean | 155.1 | 17.6 | 476.7 | 30.9 | 1597.6 | 28.3 | 350.7 | 26.6 | 34.8 | 11.1 | 1518.7 | 26.2 | 1249.7 | 30.8 | 88.9 | 15.5 | 89.4 | 4.6 | 1739.1 | 34.7 | 113.9 | 6.9 |
| 3-season mean^¥^ | 253.0 | 28.8 | 439.4 | 28.7 | 1712.6 | 29.7 | 447.2 | 34.2 | 36.1 | 11.7 | 1461.2 | 25.3 | 1655.2 | 41.3 | 109.5 | 19.4 | 139.2 | 7.2 | 2358.7 | 47.6 | 140.2 | 8.6 |

PM=Piedomont; VA=Valle d’Aosta; LO=Lombardy; BZ=Bolzano; TR=trento; VE=Veneto; FVG=Friuli Venezia Giulia; LG=Liguria; ER=Emilia Romagna; TS=Tuscany; UM=Umbria; MA=Marche; LZ=Lazio; AB=Abruzzo; MO=Molise; CA=Campania; PU=Puglia; BA=Basilizata; CB=Calabria; SI=Sicily; SA=Sardinia

*Each season extends from week 27 of one year to week 26 of the following year

§ Every 100,000 inhabitants

^¥^Includes the most recent seasons, namely 2016/17, 2017/18, and 2018/19

**Table A12.** Seasonal mean of incidence of ILI per 1000 patients among population aged 65 years and over, by region

| Season* | PM | VA | LO | BZ | TR | VE | FVG | LG | ER | TS | UM | MA | LZ | AB | MO | CA | PU | BA | CB | SI | SA |
| --- | --- | --- | --- | --- | --- | --- | --- | --- | --- | --- | --- | --- | --- | --- | --- | --- | --- | --- | --- | --- | --- |
| 2008/2009 | 2.46 | 18.42 | 0.74 | 2.95 | 0.62 | 0.37 | 0.83 | 0.97 | 1.13 | 1.30 | 0.67 | 3.55 | 1.89 | 1.56 | 0.68 | 1.87 | 0.77 | 1.41 | 1.76 | 1.14 | 1.65 |
| 2009/2010 | 1.72 | 0.68 | 0.53 | 3.74 | 0.41 | 0.30 | 0.61 | 0.59 | 0.76 | 0.95 | 0.21 | 1.88 | 1.37 | 1.49 | 1.08 | 1.46 | 0.52 | 0.96 | 0.97 | 1.05 | 0.93 |
| 2010/2011 | 1.98 | 13.06 | 0.63 | 0.37 | 0.78 | 0.75 | 0.42 | 0.88 | 0.90 | 1.14 | 0.61 | 2.32 | 1.34 | 0.95 | 0.77 | 2.51 | 0.62 | 1.61 | 0.84 | 1.12 | 0.38 |
| 2011/2012 | 2.69 | 6.10 | 1.09 | 0.67 | 1.97 | 1.37 | 0.89 | 0.90 | 1.37 | 1.60 | 0.97 | 2.13 | 1.29 | 2.09 | 1.67 | 2.06 | 0.93 | 2.54 | 1.17 | 2.07 | 2.79 |
| 2012/2013 | 2.08 | 3.94 | 0.87 | 0.67 | 1.79 | 0.95 | 0.88 | 1.08 | 1.40 | 1.28 | 1.13 | 3.62 | 1.33 | 2.71 | 1.92 | 2.71 | 0.85 | 2.60 | 1.08 | 1.76 | 2.09 |
| 2013/2014 | 1.40 | 3.44 | 0.69 | 0.71 | 1.39 | 0.61 | 0.82 | 0.95 | 1.32 | 0.88 | 1.26 | 2.72 | 1.10 | 1.31 | 2.80 | 2.75 | 0.72 | 3.24 | 1.50 | 1.69 | 2.14 |
| 2014/2015 | 2.54 | . | 1.20 | 1.57 | 2.45 | 1.20 | 1.49 | 1.63 | 2.14 | 1.66 | 2.24 | 4.79 | 2.13 | 2.66 | 1.93 | 3.75 | 1.37 | 5.49 | 3.11 | 1.69 | 3.28 |
| 2015/2016 | 1.62 | . | 0.77 | 0.98 | 1.57 | 1.02 | 1.07 | 0.80 | 1.17 | 0.77 | 0.90 | 2.69 | 1.40 | 1.88 | 0.81 | 2.67 | 0.67 | 7.32 | 3.22 | 0.49 | 2.11 |
| 2016/2017 | 2.60 | 3.45 | 1.39 | 1.57 | 3.19 | 1.62 | 1.94 | 1.80 | 2.31 | 1.74 | 2.53 | 4.40 | 2.29 | 2.08 | 1.56 | 3.13 | 1.10 | 5.37 | . | 2.02 | 1.97 |
| 2017/2018 | 2.80 | 2.88 | 2.67 | 1.60 | 3.73 | 1.58 | 1.70 | 2.91 | 1.90 | 2.28 | 1.88 | 4.12 | 2.28 | 4.68 | 5.09 | 3.07 | 2.92 | 11.48 | . | 4.37 | 2.72 |
| 2018/2019 | 2.15 | 2.72 | 2.22 | 1.28 | 2.63 | 1.37 | 1.64 | 2.11 | 1.46 | 1.85 | 1.34 | 3.22 | 1.94 | 3.76 | 2.63 | 2.95 | 2.01 | 2.77 | 3.58 | 4.64 | 1.43 |
| *11-season mean* | *2.18* | *6.08* | *1.16* | *1.46* | *1.87* | *1.01* | *1.12* | *1.33* | *1.44* | *1.40* | *1.25* | *3.22* | *1.67* | *2.29* | *1.90* | *2.63* | *1.14* | *4.07* | *1.91* | *2.00* | *1.95* |
| *3-season mean^§^* | *2.5* | *3.0* | *2.1* | *1.5* | *3.2* | *1.5* | *1.8* | *2.3* | *1.9* | *2.0* | *1.9* | *3.9* | *2.2* | *3.5* | *3.1* | *3.1* | *2.0* | *6.5* | *3.6* | *3.7* | *2.0* |

*Each season extends from week 27 of one year to week 26 of the following year

*^§^*Includes the most recent seasons, namely 2016/17, 2017/18, and 2018/19

**Table A13.** Rate per 100,000 people of hospitalizations with primary code 487 among people aged 65+, by region

| Season* | PM | VA | LO | BZ | TR | VE | FVG | LG | ER | TS | UM | MA | LZ | AB | MO | CA | PU | BA | CB | SI | SA |
| --- | --- | --- | --- | --- | --- | --- | --- | --- | --- | --- | --- | --- | --- | --- | --- | --- | --- | --- | --- | --- | --- |
| 2008/2009 | 2.6 | 0 | 4.2 | 40.5 | 6.1 | 8.6 | 7.1 | 3.3 | 10.8 | 4.7 | 12.2 | 5.5 | 9.8 | 12.1 | 4.3 | 3.8 | 4.5 | 5.1 | 7.9 | 4.9 | 23.6 |
| 2009/2010 | 2.6 | 7.6 | 5.7 | 19.2 | 2 | 6.6 | 5.6 | 3 | 8.7 | 6.6 | 15.5 | 12.6 | 9.8 | 12.4 | 2.9 | 9.3 | 12 | 3.4 | 9.2 | 6.2 | 11.7 |
| 2010/2011 | 2.3 | 0 | 4.2 | 34.4 | 2 | 6.9 | 6.6 | 2.6 | 10.6 | 5.4 | 15.9 | 11.1 | 4.9 | 7 | 1.4 | 2 | 7.4 | 2.5 | 5.4 | 9.1 | 12.7 |
| 2011/2012 | 3.5 | 11.1 | 4.9 | 20.7 | 2.9 | 6.4 | 5.8 | 3 | 8.5 | 7.9 | 8.6 | 2.3 | 4.3 | 10.1 | 1.4 | 1.7 | 6.9 | 2.5 | 2.7 | 6.4 | 15.5 |
| 2012/2013 | 2.3 | 18.2 | 5.2 | 25.5 | 6.6 | 7.4 | 6.7 | 2.7 | 13.5 | 6.3 | 9.9 | 3.4 | 3.2 | 11.4 | 4.3 | 2.9 | 8.2 | 4.2 | 3.4 | 5.2 | 7.1 |
| 2013/2014 | 2.3 | 3.6 | 3.1 | 12.5 | 8.3 | 3.2 | 8 | 2.3 | 7 | 2.2 | 9.8 | 3 | 3.3 | 4.1 | 2.8 | 1.6 | 5.2 | 1.7 | 3.1 | 3.1 | 11 |
| 2014/2015 | 4 | 24.5 | 9.1 | 43 | 12.6 | 16.7 | 3.6 | 7.7 | 16.7 | 6.3 | 15.6 | 6.3 | 2.5 | 4.7 | 4.2 | 3.6 | 8.1 | 3.2 | 7.4 | 2.7 | 12.4 |
| 2015/2016 | 2.8 | 3.5 | 4.7 | 14.1 | 15.9 | 10.1 | 2.9 | 1.8 | 10.6 | 2.7 | 10.9 | 1.6 | 1.5 | 4.6 | 2.7 | 1.9 | 3.4 | 0 | 4.5 | 1.9 | 6.4 |
| 2016/2017 | 3.4 | 13.7 | 13.7 | 46.7 | 9.6 | 18.6 | 6.4 | 7.9 | 29 | 7.1 | 12.7 | 3.2 | 4 | 4.9 | 1.4 | 2.9 | 7 | 3.2 | 3.7 | 2.5 | 11.9 |
| 2017/2018 | 8.1 | 13.5 | 14.9 | 73.5 | 7.7 | 18 | 16.6 | 15.7 | 35.7 | 7.4 | 14 | 5.3 | 3.6 | 12.4 | 4.1 | 1.7 | 7.5 | 2.4 | 4.2 | 3.2 | 19.5 |
| 2018/2019 | 13.3 | 0 | 14.4 | 97.6 | 23.5 | 23.3 | 35.4 | 9.8 | 37.5 | 11.2 | 8.5 | 4.8 | 4.6 | 7.1 | 2.7 | 1.8 | 8.6 | 0.8 | 1.7 | 3.5 | 16.5 |
| *11-season mean* | *4.3* | *8.7* | *7.6* | *38.9* | *8.8* | *11.4* | *9.5* | *5.4* | *17.1* | *6.2* | *12.1* | *5.4* | *4.7* | *8.3* | *2.9* | *3* | *7.2* | *2.6* | *4.8* | *4.4* | *13.5* |
| *3-season mean^§^* | *8.3* | *9.1* | *14.3* | *72.6* | *13.6* | *20.0* | *19.5* | *11.1* | *34.1* | *8.6* | *11.7* | *4.4* | *4.1* | *8.1* | *2.7* | *2.1* | *7.7* | *2.1* | *3.2* | *3.1* | *16.0* |

*Each season extends from week 27 of one year to week 26 of the following year

*^§^*Includes the most recent seasons, namely 2016/17, 2017/18, and 2018/19

**Figure A1.** Average rates per 100,000 people of excess hospitalization associated with influenza from seasons 2008/09 to 2018/19, by region. All ages

**Table A14**. Seasonal costs of influenza and influenza-associated hospitalisations. Season 2008/09-2018/19, Italy

| Season* | Hospitalisations with primary diagnosis influenza (N) | Costs of hosp. with primary diagnosis influenza (€) | Estimated hospitalisations associated with influenza (N) | Costs for hosp. associated with influenza | | Total hospitalisations for influenza (N) | | Total costs | | |  |
| --- | --- | --- | --- | --- | --- | --- | --- | --- | --- | --- | --- |
|  |  |  |  | Prudential estimate (€) | Wide scope estimate (€) |  |  | Prudential estimate (€) | | Wide scope estimate (€) |  |
| 2008/09 | 3,404 | 5,298,818 | 16,503 | 35,628,192 | 85,688,076 | 19,907 | 40,927,010 | | 103,363,108 | | |
| 2009/10 | 9,054 | 23,228,158 | 20,613 | 44,503,018 | 107,032,598 | 29,667 | 67,731,176 | | 154,044,859 | | |
| 2010/11 | 4,022 | 14,656,090 | 22,797 | 49,217,533 | 118,371,307 | 26,819 | 63,873,623 | | 139,255,261 | | |
| 2011/12 | 2,631 | 4,436,365 | 18,966 | 40,946,136 | 98,478,070 | 21,597 | 45,382,501 | | 112,139,353 | | |
| 2012/13 | 3,449 | 8,949,985 | 23,241 | 50,177,190 | 120,679,343 | 26,690 | 59,127,175 | | 138,588,034 | | |
| 2013/14 | 2,447 | 5,037,126 | 16,849 | 36,375,623 | 87,485,695 | 19,296 | 41,412,749 | | 100,191,572 | | |
| 2014/15 | 3,807 | 16,682,681 | 22,885 | 49,408,385 | 118,830,318 | 26,692 | 66,091,066 | | 138,597,899 | | |
| 2015/16 | 2,482 | 6,445,774 | 17,203 | 37,140,542 | 89,325,373 | 19,685 | 43,586,316 | | 102,212,985 | | |
| 2016/17 | 3,128 | 7,013,613 | 19,073 | 41,177,144 | 99,033,660 | 22,201 | 48,190,757 | | 115,275,581 | | |
| 2017/18 | 4,596 | 17,901,757 | 30,556 | 65,968,235 | 158,657,814 | 35,152 | 83,869,992 | | 182,522,222 | | |
| 2018/19 | 4,642 | 17,380,886 | 28,211 | 60,907,207 | 146,485,720 | 32,853 | 78,288,093 | | 170,588,980 | | |
| *Total* | 43,662 | 127,031,253 | 236,896 | 511,449,206 | 1,230,067,974 | 280,558 | 638,480,459 | | 1,456,779,853 | | |
| 11-season mean | 3,969 | 11,548,296 | 21,536 | 46,495,382 | 111,824,361 | 25,505 | 58,043,678 | | 132,434,532 | | |
| 3-season mean*^§^* | 4122.0 | 14098752.0 | 25946.7 | 56017528.7 | 134725731.3 | 30068.7 | 70116280.7 | | 156128927.7 | | |

*Each season extends from week 27 of one year to week 26 of the following year

*^§^*Includes the most recent seasons, namely 2016/17, 2017/18, and 2018/19

**Table A15.** In hospital mortality from seasons 2008/09 to 2018/19, by different groups of diagnoses at hospital admission. All ages

| Season* | Influenza (Primary diagnosis) | Influenza (Primary or secondary diagnosis) | Influenza and pneumonia  (Primary diagnosis) | Respiratory diseases  (Primary diagnosis) | Cardiorespiratory diseases  (Primary diagnosis) | All considered ICD9 diseases  (Primary diagnosis) |
| --- | --- | --- | --- | --- | --- | --- |
| 2008/09 | 15 | 57 | 7,672 | 34,532 | 76,749 | 78,199 |
| 2009/10 | 76 | 204 | 8,168 | 36,052 | 77,817 | 79,395 |
| 2010/11 | 63 | 174 | 8,473 | 37,424 | 78,186 | 79,729 |
| 2011/12 | 16 | 62 | 9,244 | 39,886 | 81,486 | 82,891 |
| 2012/13 | 23 | 83 | 8,984 | 39,485 | 78,697 | 79,940 |
| 2013/14 | 7 | 40 | 8,994 | 39,577 | 78,119 | 79,254 |
| 2014/15 | 89 | 289 | 10,317 | 44,419 | 85,110 | 86,466 |
| 2015/16 | 25 | 80 | 10,304 | 43,879 | 83,062 | 84,301 |
| 2016/17 | 57 | 205 | 11,360 | 47,622 | 87,072 | 88,407 |
| 2017/18 | 65 | 310 | 11,196 | 47,651 | 84,805 | 86,094 |
| 2018/19 | 117 | 491 | 11,263 | 49,377 | 85,407 | 86,741 |
| *Total* | *558* | *2,007* | *110,455* | *480,070* | *931,696* | *947,026* |
| 11-season mean | 43.6 | 150.4 | 9471.2 | 41052.7 | 81110.3 | 82467.6 |
| 3-season mean*^§^* | 79.7 | 335.3 | 11273.0 | 48216.7 | 85761.3 | 87080.7 |

*Each season extends from week 27 of one year to week 26 of the following year

*^§^*Includes the most recent seasons, namely 2016/17, 2017/18, and 2018/19

**Table A16**. In hospital mortality from seasons 2008/09 to 2018/19, by region. All ages

| Season* | PM | VA | LO | BZ | TR | VE | FVG | LG | ER | TS | UM | MA | LZ | AB | MO | CA | PU | BA | CB | SI | SA |
| --- | --- | --- | --- | --- | --- | --- | --- | --- | --- | --- | --- | --- | --- | --- | --- | --- | --- | --- | --- | --- | --- |
| **2008/2009** |  |  |  |  |  |  |  |  |  |  |  |  |  |  |  |  |  |  |  |  |  |
| Influenza | 1 | 0 | 1 | 0 | 0 | 2 | 1 | 0 | 2 | 2 | 0 | 1 | 5 | 0 | 0 | 0 | 0 | 0 | 0 | 0 | 0 |
| All ICD9 considered | 7,399 | 205 | 11,461 | 664 | 671 | 7,721 | 2,673 | 3,534 | 9,102 | 5,781 | 1,066 | 2,820 | 7,946 | 1,888 | 438 | 4,215 | 3,390 | 511 | 1,606 | 3,368 | 1,725 |
| **2009/2010** |  |  |  |  |  |  |  |  |  |  |  |  |  |  |  |  |  |  |  |  |  |
| Influenza | 7 | 0 | 15 | 0 | 1 | 2 | 2 | 3 | 5 | 2 | 0 | 0 | 4 | 4 | 2 | 4 | 3 | 0 | 6 | 14 | 2 |
| All ICD9 considered | 7,430 | 237 | 11,374 | 700 | 632 | 7,692 | 2,536 | 3,556 | 9,185 | 6,093 | 1,161 | 2,867 | 8,279 | 1,832 | 400 | 4,278 | 3,516 | 616 | 1,550 | 3,516 | 1,869 |
| **2010/2011** |  |  |  |  |  |  |  |  |  |  |  |  |  |  |  |  |  |  |  |  |  |
| Influenza | 7 | 0 | 24 | 1 | 0 | 4 | 1 | 0 | 2 | 10 | 0 | 2 | 3 | 0 | 0 | 3 | 2 | 0 | 2 | 2 | 0 |
| All ICD9 considered | 7,101 | 194 | 11,417 | 530 | 581 | 7,724 | 2,551 | 3,582 | 8,835 | 6,244 | 1,229 | 2,864 | 8,539 | 1,925 | 470 | 4,469 | 3,633 | 634 | 1,680 | 3,615 | 1,849 |
| **2011/2012** |  |  |  |  |  |  |  |  |  |  |  |  |  |  |  |  |  |  |  |  |  |
| Influenza | 0 | 0 | 1 | 1 | 0 | 1 | 0 | 0 | 1 | 6 | 0 | 0 | 2 | 1 | 0 | 0 | 2 | 0 | 0 | 0 | 1 |
| All ICD9 considered | 7,382 | 216 | 11,614 | 602 | 737 | 8,130 | 2,652 | 3,508 | 9,305 | 6,591 | 1,227 | 2,904 | 8,584 | 2,160 | 485 | 4,592 | 3,789 | 692 | 1,722 | 4,073 | 1,910 |
| **2012/2013** |  |  |  |  |  |  |  |  |  |  |  |  |  |  |  |  |  |  |  |  |  |
| Influenza | 3 | 0 | 7 | 0 | 0 | 1 | 0 | 0 | 5 | 1 | 0 | 0 | 3 | 2 | 1 | 0 | 0 | 0 | 0 | 0 | 0 |
| All ICD9 considered | 7,286 | 234 | 11,044 | 584 | 588 | 7,591 | 2,510 | 3,450 | 8,793 | 6,066 | 1,225 | 2,762 | 8,318 | 2,145 | 492 | 4,778 | 3,670 | 745 | 1,763 | 4,008 | 1,865 |
| **2013/2014** |  |  |  |  |  |  |  |  |  |  |  |  |  |  |  |  |  |  |  |  |  |
| Influenza | 0 | 0 | 1 | 0 | 0 | 1 | 0 | 1 | 1 | 0 | 0 | 0 | 2 | 0 | 0 | 0 | 0 | 0 | 0 | 1 | 0 |
| All ICD9 considered | 7,048 | 232 | 10,725 | 591 | 619 | 7,403 | 2,394 | 3,199 | 8,592 | 5,786 | 1,355 | 2,880 | 8,261 | 2,175 | 469 | 4,816 | 4,008 | 778 | 1,897 | 4,104 | 1,915 |
| **2014/2015** |  |  |  |  |  |  |  |  |  |  |  |  |  |  |  |  |  |  |  |  |  |
| Influenza | 8 | 2 | 21 | 2 | 0 | 21 | 3 | 0 | 10 | 5 | 2 | 4 | 1 | 0 | 0 | 4 | 2 | 0 | 0 | 4 | 0 |
| All ICD9 considered | 7,742 | 259 | 12,038 | 627 | 627 | 8,009 | 2,442 | 3,491 | 9,299 | 6,319 | 1,417 | 3,063 | 8,890 | 2,335 | 538 | 5,350 | 4,437 | 840 | 1,971 | 4,636 | 2,047 |
| **2015/2016** |  |  |  |  |  |  |  |  |  |  |  |  |  |  |  |  |  |  |  |  |  |
| Influenza | 2 | 0 | 5 | 0 | 0 | 3 | 0 | 0 | 3 | 2 | 2 | 0 | 0 | 1 | 1 | 0 | 1 | 1 | 1 | 3 | 0 |
| All ICD9 considered | 7,298 | 238 | 11,619 | 573 | 598 | 8,045 | 2,493 | 3,265 | 9,070 | 6,059 | 1,341 | 2,869 | 8,644 | 2,313 | 551 | 5,381 | 4,521 | 865 | 1,899 | 4,585 | 2,049 |
| **2016/2017** |  |  |  |  |  |  |  |  |  |  |  |  |  |  |  |  |  |  |  |  |  |
| Influenza | 4 | 0 | 11 | 1 | 0 | 9 | 3 | 1 | 16 | 5 | 0 | 0 | 2 | 0 | 0 | 1 | 2 | 0 | 0 | 2 | 0 |
| All ICD9 considered | 7,444 | 269 | 12,312 | 635 | 633 | 8,118 | 2,611 | 3,450 | 9,278 | 6,418 | 1,372 | 2,966 | 9,535 | 2,513 | 504 | 5,693 | 4,744 | 977 | 2,050 | 4,785 | 2,043 |
| **2017/2018** |  |  |  |  |  |  |  |  |  |  |  |  |  |  |  |  |  |  |  |  |  |
| Influenza | 9 | 0 | 21 | 1 | 1 | 5 | 1 | 1 | 10 | 3 | 1 | 0 | 3 | 0 | 0 | 0 | 3 | 0 | 0 | 4 | 2 |
| All ICD9 considered | 7,364 | 294 | 12,034 | 660 | 546 | 7,959 | 2,372 | 3,552 | 8,903 | 6,108 | 1,354 | 2,756 | 9,230 | 2,378 | 261 | 5,488 | 4,658 | 867 | 2,054 | 5,034 | 2,157 |
| **2018/2019** |  |  |  |  |  |  |  |  |  |  |  |  |  |  |  |  |  |  |  |  |  |
| Influenza | 17 | 0 | 22 | 1 | 2 | 19 | 10 | 1 | 21 | 6 | 1 | 2 | 8 | 0 | 0 | 2 | 1 | 0 | 1 | 3 | 0 |
| All ICD9 considered | 7,288 | 280 | 11,929 | 632 | 563 | 7,938 | 2,452 | 3,361 | 9,200 | 6,012 | 1,296 | 2,979 | 9,295 | 2,325 | 296 | 5,637 | 4,839 | 942 | 2,277 | 4,888 | 2,195 |
| **Total** |  |  |  |  |  |  |  |  |  |  |  |  |  |  |  |  |  |  |  |  |  |
| Influenza | 58 | 2 | 129 | 7 | 4 | 68 | 21 | 7 | 76 | 42 | 6 | 9 | 33 | 8 | 4 | 14 | 16 | 1 | 10 | 33 | 5 |
| All ICD9 considered | 80782 | 2658 | 127567 | 6798 | 6795 | 86330 | 27686 | 37948 | 99562 | 67477 | 14043 | 31730 | 95521 | 23989 | 4904 | 54697 | 45205 | 8467 | 20469 | 46612 | 21624 |
| **Mean** |  |  |  |  |  |  |  |  |  |  |  |  |  |  |  |  |  |  |  |  |  |
| Influenza | 5.3 | 0.2 | 11.7 | 0.6 | 0.4 | 6.2 | 1.9 | 0.6 | 6.9 | 3.8 | 0.5 | 0.8 | 3 | 0.7 | 0.4 | 1.3 | 1.5 | 0.1 | 0.9 | 3 | 0.5 |
| All ICD9 considered | 7343.8 | 241.6 | 11597 | 618 | 617.7 | 7848.2 | 2516.9 | 3449.8 | 9051.1 | 6134.3 | 1276.6 | 2884.5 | 8683.7 | 2180.8 | 445.8 | 4972.5 | 4109.5 | 769.7 | 1860.8 | 4237.5 | 1965.8 |

PM=Piedomont; VA=Valle d’Aosta; LO=Lombardy; BZ=Bolzano; TR=trento; VE=Veneto; FVG=Friuli Venezia Giulia; LG=Liguria; ER=Emilia Romagna; TS=Tuscany; UM=Umbria; MA=Marche; LZ=Lazio; AB=Abruzzo; MO=Molise; CA=Campania; PU=Puglia; BA=Basilicata; CB=Calabria; SI=Sicily; SA=Sardinia

*Each season extends from week 27 of one year to week 26 of the following year

**Table A17.** Estimates of numbers and rates per 100,000 people of excess in-hospital mortality associated with influenza, by region and season. All ages

| Season* | PM | | VA | | LO | | BZ | | TR | | VE | | FVG | | LG | | ER | | TS | |
| --- | --- | --- | --- | --- | --- | --- | --- | --- | --- | --- | --- | --- | --- | --- | --- | --- | --- | --- | --- | --- |
|  | N | Rate^¥^ | N | Rate^¥^ | N | Rate^¥^ | N | Rate^¥^ | N | Rate^¥^ | N | Rate^¥^ | N | Rate^¥^ | N | Rate^¥^ | N | Rate^¥^ | N | Rate^¥^ |
| 2008/2009 | 284.9 | 6.5 | 7.0 | 5.5 | 419.5 | 4.4 | 21.6 | 4.4 | 26.0 | 5.1 | 292.6 | 6.1 | 67.7 | 5.5 | 129.2 | 8.1 | 264.6 | 6.2 | 173.6 | 4.7 |
| 2009/2010 | 354.5 | 8.1 | 8.6 | 6.8 | 520.3 | 5.4 | 26.7 | 5.4 | 31.4 | 6.0 | 363.9 | 7.5 | 81.3 | 6.6 | 158.7 | 10.0 | 326.2 | 7.5 | 213.3 | 5.8 |
| 2010/2011 | 439.3 | 10.0 | 11.4 | 8.9 | 655.9 | 6.8 | 33.3 | 6.6 | 39.0 | 7.4 | 455.6 | 9.4 | 103.1 | 8.4 | 195.8 | 12.3 | 408.0 | 9.4 | 267.2 | 7.2 |
| 2011/2012 | 346.2 | 7.8 | 9.3 | 7.3 | 522.1 | 5.3 | 26.3 | 5.2 | 30.2 | 5.7 | 361.1 | 7.4 | 81.2 | 6.6 | 155.4 | 9.8 | 322.7 | 7.4 | 211.5 | 5.7 |
| 2012/2013 | 422.7 | 9.6 | 11.9 | 9.3 | 639.0 | 6.5 | 32.0 | 6.3 | 36.4 | 6.8 | 441.3 | 9.0 | 97.2 | 7.9 | 187.7 | 11.8 | 392.0 | 8.9 | 257.2 | 6.9 |
| 2013/2014 | 300.3 | 6.8 | 8.8 | 6.8 | 454.2 | 4.6 | 22.9 | 4.5 | 25.8 | 4.8 | 313.5 | 6.4 | 68.3 | 5.6 | 131.4 | 8.3 | 280.7 | 6.3 | 184.9 | 4.9 |
| 2014/2015 | 423.9 | 9.6 | 12.7 | 9.9 | 646.7 | 6.5 | 32.177 | 6.2 | 35.7 | 6.6 | 445.1 | 9.1 | 95.787 | 7.8 | 185.1 | 11.8 | 394.3 | 8.9 | 259.2 | 6.9 |
| 2015/2016 | 328.22 | 7.5 | 10.3 | 8.1 | 503.08 | 5.1 | 24.959 | 4.8 | 27.452 | 5.1 | 345.66 | 7.1 | 74.051 | 6.1 | 142.5 | 9.1 | 306.7 | 6.9 | 202.7 | 5.4 |
| 2016/2017 | 381.01 | 8.7 | 12.1 | 9.6 | 592.16 | 5.9 | 28.91 | 5.5 | 30.642 | 5.7 | 403.52 | 8.3 | 85.97 | 7.1 | 165.4 | 10.6 | 355.2 | 8.0 | 232.4 | 6.2 |
| 2017/2018 | 585.70 | 13.4 | 19.1 | 15.1 | 912.79 | 9.1 | 44.262 | 8.4 | 46.37 | 8.6 | 620.65 | 12.7 | 128.97 | 10.6 | 252.6 | 16.3 | 539.63 | 12.1 | 353.54 | 9.5 |
| 2018/2019 | 498.9 | 11.5 | 16.8 | 13.4 | 783.0 | 7.8 | 37.8 | 7.2 | 39.3 | 7.2 | 531.1 | 10.9 | 110.3 | 9.1 | 214.3 | 13.9 | 463.2 | 10.4 | 304.17 | 8.2 |
| Total | 4365.6 |  | 128.0 |  | 6648.7 |  | 331.0 |  | 368.1 |  | 4573.9 |  | 993.9 |  | 1918.2 |  | 4053.1 |  | 2659.8 |  |
| 11-season mean | 396.9 | 9.0 | 11.6 | 9.2 | 604.4 | 6.1 | 30.1 | 5.9 | 33.5 | 6.3 | 415.8 | 8.5 | 90.4 | 7.4 | 174.4 | 11.1 | 368.5 | 8.4 | 241.8 | 6.5 |
| 3-season mean*^§^* | 488.5 | 11.2 | 16.0 | 12.7 | 762.7 | 7.6 | 37.0 | 7.0 | 38.8 | 7.2 | 518.4 | 10.6 | 108.4 | 8.9 | 210.8 | 13.6 | 452.7 | 10.2 | 296.7 | 8.0 |

| Season* | UM | | MA | | LZ | | AB | | MO | | CA | | PU | | BA | | CB | | SI | | SA | |
| --- | --- | --- | --- | --- | --- | --- | --- | --- | --- | --- | --- | --- | --- | --- | --- | --- | --- | --- | --- | --- | --- | --- |
|  | N | Rate^¥^ | N | Rate^¥^ | N | Rate^¥^ | N | Rate^¥^ | N | Rate^¥^ | N | Rate^¥^ | N | Rate^¥^ | N | Rate^¥^ | N | Rate^¥^ | N | Rate^¥^ | N | Rate^¥^ |
| 2008/2009 | 33.1 | 3.8 | 98.3 | 6.4 | 294.9 | 5.4 | 61.4 | 4.7 | 2.0 | 0.6 | 166.9 | 2.9 | 117.1 | 2.9 | 16.5 | 2.8 | 44.3 | 2.2 | 109.6 | 2.2 | 64.5 | 3.9 |
| 2009/2010 | 41.2 | 4.7 | 120.9 | 7.8 | 368.7 | 6.7 | 77.1 | 5.8 | 2.3 | 0.7 | 213.7 | 3.7 | 150.2 | 3.7 | 20.7 | 3.5 | 55.4 | 2.8 | 137.3 | 2.7 | 78.6 | 4.8 |
| 2010/2011 | 52.8 | 6.0 | 151.6 | 9.8 | 467.3 | 8.4 | 99.5 | 7.5 | 2.9 | 0.9 | 274.9 | 4.7 | 194.3 | 4.7 | 27.8 | 4.8 | 72.4 | 3.7 | 182.0 | 3.6 | 102.8 | 6.2 |
| 2011/2012 | 42.6 | 4.8 | 119.7 | 7.7 | 374.9 | 6.7 | 80.5 | 6.0 | 2.3 | 0.7 | 222.9 | 3.8 | 159.2 | 3.9 | 23.7 | 4.1 | 59.5 | 3.0 | 149.3 | 3.0 | 83.2 | 5.0 |
| 2012/2013 | 52.8 | 5.9 | 146.9 | 9.5 | 463.2 | 8.2 | 101.3 | 7.6 | 2.7 | 0.9 | 281.7 | 4.8 | 201.5 | 4.9 | 30.2 | 5.2 | 74.8 | 3.8 | 190.5 | 3.8 | 103.7 | 6.3 |
| 2013/2014 | 38.7 | 4.3 | 106.2 | 6.8 | 333.8 | 5.9 | 75.4 | 5.7 | 2.0 | 0.6 | 208.2 | 3.6 | 149.6 | 3.7 | 22.8 | 4.0 | 55.6 | 2.8 | 143.4 | 2.8 | 75.5 | 4.6 |
| 2014/2015 | 55.2 | 6.2 | 148.8 | 9.6 | 478.1 | 8.3 | 108.4 | 8.2 | 2.6 | 0.8 | 302.2 | 5.2 | 218.4 | 5.4 | 33.9 | 5.9 | 80.6 | 4.1 | 208.0 | 4.1 | 108.4 | 6.6 |
| 2015/2016 | 43.9 | 5.0 | 117.2 | 7.6 | 375.7 | 6.5 | 87.5 | 6.6 | 2.1 | 0.7 | 243.0 | 4.2 | 176.5 | 4.4 | 27.7 | 4.9 | 65.2 | 3.4 | 170.7 | 3.4 | 86.0 | 5.2 |
| 2016/2017 | 51.5 | 5.8 | 131.6 | 8.6 | 440.7 | 7.6 | 100.9 | 7.7 | 2.2 | 0.7 | 287.1 | 5.0 | 210.6 | 5.2 | 33.9 | 6.0 | 76.6 | 4.0 | 198.2 | 4.0 | 99.7 | 6.1 |
| 2017/2018 | 79.7 | 9.1 | 202.8 | 13.3 | 683.5 | 11.8 | 158.37 | 12.1 | 3.3 | 1.1 | 457.1 | 7.9 | 334.3 | 8.3 | 53.5 | 9.5 | 120.3 | 6.2 | 316.8 | 6.4 | 156 | 9.5 |
| 2018/2019 | 69.9 | 8.0 | 175.0 | 11.5 | 594.2 | 10.3 | 140.1 | 10.8 | 2.8 | 0.9 | 402.8 | 7.0 | 297.5 | 7.5 | 49.0 | 8.7 | 107.6 | 5.6 | 283.0 | 5.7 | 137.3 | 8.4 |
| Total | 561.3 |  | 1519.0 |  | 4874.8 |  | 1090.6 |  | 27.3 |  | 3060.4 |  | 2209.3 |  | 339.6 |  | 812.1 |  | 2088.9 |  | 1095.7 |  |
| 11-season mean | 51.0 | 5.8 | 138.1 | 9.0 | 443.2 | 7.8 | 99.1 | 7.5 | 2.5 | 0.8 | 278.2 | 4.8 | 200.8 | 5.0 | 30.9 | 5.4 | 73.8 | 3.8 | 189.9 | 3.8 | 99.6 | 6.1 |
| 3-season mean*^§^* | 67.0 | 7.6 | 169.8 | 11.1 | 572.8 | 9.9 | 133.1 | 10.2 | 2.8 | 0.9 | 382.3 | 6.6 | 280.8 | 7.0 | 45.5 | 8.1 | 101.5 | 5.3 | 266.0 | 5.4 | 131.0 | 8.0 |

PM=Piedomont; VA=Valle d’Aosta; LO=Lombardy; BZ=Bolzano; TR=trento; VE=Veneto; FVG=Friuli Venezia Giulia; LG=Liguria; ER=Emilia Romagna; TS=Tuscany; UM=Umbria; MA=Marche; LZ=Lazio; AB=Abruzzo; MO=Molise; CA=Campania; PU=Puglia; BA=Basilizata; CB=Calabria; SI=Sicily; SA=Sardinia

*Each season extends from week 27 of one year to week 26 of the following year

^¥^ Every 100,000 inhabitants

*^§^*Includes the most recent seasons, namely 2016/17, 2017/18, and 2018/19
